# Precision mapping of snail habitat provides a powerful indicator of human schistosomiasis transmission

**DOI:** 10.1073/pnas.1903698116

**Published:** 2019-10-28

**Authors:** Chelsea L. Wood, Susanne H. Sokolow, Isabel J. Jones, Andrew J. Chamberlin, Kevin D. Lafferty, Armand M. Kuris, Merlijn Jocque, Skylar Hopkins, Grant Adams, Julia C. Buck, Andrea J. Lund, Ana E. Garcia-Vedrenne, Evan Fiorenza, Jason R. Rohr, Fiona Allan, Bonnie Webster, Muriel Rabone, Joanne P. Webster, Lydie Bandagny, Raphaël Ndione, Simon Senghor, Anne-Marie Schacht, Nicolas Jouanard, Gilles Riveau, Giulio A. De Leo

**Affiliations:** ^a^School of Aquatic and Fishery Sciences, University of Washington, Seattle, WA 98195;; ^b^Hopkins Marine Station, Stanford University, Pacific Grove, CA 93950;; ^c^Western Ecological Research Center, United States Geological Survey, Santa Barbara, CA 93106;; ^d^Marine Science Institute, University of California, Santa Barbara, CA 93106;; ^e^Aquatic and Terrestrial Ecology, Royal Belgian Institute of Natural Sciences, 1000 Brussels, Belgium;; ^f^Department of Biological Sciences, Virginia Polytechnic Institute and State University, Blacksburg, VA 24060;; ^g^Department of Biology and Marine Biology, University of North Carolina Wilmington, Wilmington, NC 28403;; ^h^Emmett Interdisciplinary Program in Environment and Resources, Stanford University, Stanford, CA 94305;; ^i^Department of Ecology and Evolutionary Biology, University of California, Los Angeles, CA 90095;; ^j^Department of Biological Sciences, Environmental Change Initiative, Eck Institute of Global Health, University of Notre Dame, Notre Dame, IN 46556;; ^k^Wolfson Wellcome Biomedical Laboratories, Department of Life Sciences, Natural History Museum, London SW7 5BD, United Kingdom;; ^l^London Centre for Neglected Tropical Disease Research, Imperial College London School of Public Health, London W2 1PG, United Kingdom;; ^m^Centre for Emerging, Endemic, and Exotic Diseases, Department of Pathology and Population Sciences, Royal Veterinary College, University of London, London NW1 0TU, United Kingdom;; ^n^Biomedical Research Center Espoir Pour La Santé, BP 226 Saint-Louis, Senegal;; ^o^Station d’Innovation Aquacole, BP 524 Saint-Louis, Senegal

**Keywords:** bilharzia, ecological levers for infectious disease control, snail control, spatial ecology, urogenital schistosomiasis

## Abstract

Recently, the World Health Organization recognized that efforts to interrupt schistosomiasis transmission through mass drug administration have been ineffective in some regions; one of their new recommended strategies for global schistosomiasis control emphasizes targeting the freshwater snails that transmit schistosome parasites. We sought to identify robust indicators that would enable precision targeting of these snails. At the site of the world’s largest recorded schistosomiasis epidemic—the Lower Senegal River Basin in Senegal—intensive sampling revealed positive relationships between intermediate host snails (abundance, density, and prevalence) and human urogenital schistosomiasis reinfection (prevalence and intensity in schoolchildren after drug administration). However, we also found that snail distributions were so patchy in space and time that obtaining useful data required effort that exceeds what is feasible in standard monitoring and control campaigns. Instead, we identified several environmental proxies that were more effective than snail variables for predicting human infection: the area covered by suitable snail habitat (i.e., floating, nonemergent vegetation), the percent cover by suitable snail habitat, and size of the water contact area. Unlike snail surveys, which require hundreds of person-hours per site to conduct, habitat coverage and site area can be quickly estimated with drone or satellite imagery. This, in turn, makes possible large-scale, high-resolution estimation of human urogenital schistosomiasis risk to support targeting of both mass drug administration and snail control efforts.

## Significance

Schistosomiasis is a parasitic disease that affects ∼206 million people globally. The World Health Organization recently endorsed control of the freshwater snails that host schistosome infectious stages, and here, we show how to better target those snail control efforts. Schistosomiasis infection occurred on a local scale at our study sites in northwestern Senegal, suggesting that small-scale interventions can suppress transmission. However, snail clusters were so ephemeral that attempts to target them for removal would be inefficient. Instead, we found easy-to-measure environmental proxies that were more effective than snail variables at predicting human infections, including area of snail habitat within the site and total site area. Our work indicates that satellite- or drone-based precision mapping could efficiently identify high-transmission areas.

Before the mid-1970s, control of human schistosomiasis was achieved by targeting the freshwater snails that host the parasite’s larval stage (1, 2). However, in 1974, control efforts shifted when the new antiparasitic drug, praziquantel, made it possible to cheaply and effectively treat existing schistosomiasis infections through mass drug administration (3–5), increasing hope that schistosomiasis could be eliminated from many world regions. Unfortunately, praziquantel was not a silver bullet; because the drug cannot prevent reinfection (6–10), prevalence often returns to its baseline once drug treatment stops (11). Today, schistosomiasis is the world’s second-most important parasitic disease of humans, affecting over 200 million people (12) and causing the loss of 3 million years of healthy life annually (13). Given this persistent, high global burden, the new strategy recommended by the World Health Organization (WHO) for global schistosomiasis control is to add snail control to existing mass drug administration campaigns (1, 2, 9, 10, 14, 15). Now that the WHO has committed to snail control (10, 16, 17), the next step is to identify the most effective strategies for finding and eliminating schistosome-competent snails.

Unfortunately, the WHO’s renewed emphasis on snail control is complicated by the fact that it is surprisingly difficult to use snail distributions to predict human schistosomiasis infections. Many studies have failed to detect a correlation between intermediate-host snail abundance or infection prevalence and human infection burden (18–24). Is this because these studies used inappropriate snail sampling techniques, because infection in humans reflects the cumulative effects of many years of exposure, or because humans do not acquire infections on the local scales at which the snail surveys were conducted (18–24)? If humans do not acquire infections at local scales, then local-scale snail control is unlikely to produce reductions in local human infection burdens. If humans do acquire infections at local scales, then snail control efforts should focus on high-transmission areas identified by surveying snails, or using some environmental proxy for snail abundance. Our study sought to address the following questions: 1) Can improved, quantitative snail sampling yield correspondence between local snail presence and local human infection burden, suggesting that human infections are acquired locally and are therefore amenable to local transmission control interventions? 2) Do snails cluster in space, and if so, are those clusters sufficiently persistent in time that we can target them using snail control interventions? 3) Are there easy-to-measure environmental correlates that might effectively predict the abundance of snails and of human infections?

**Fig. 1.**
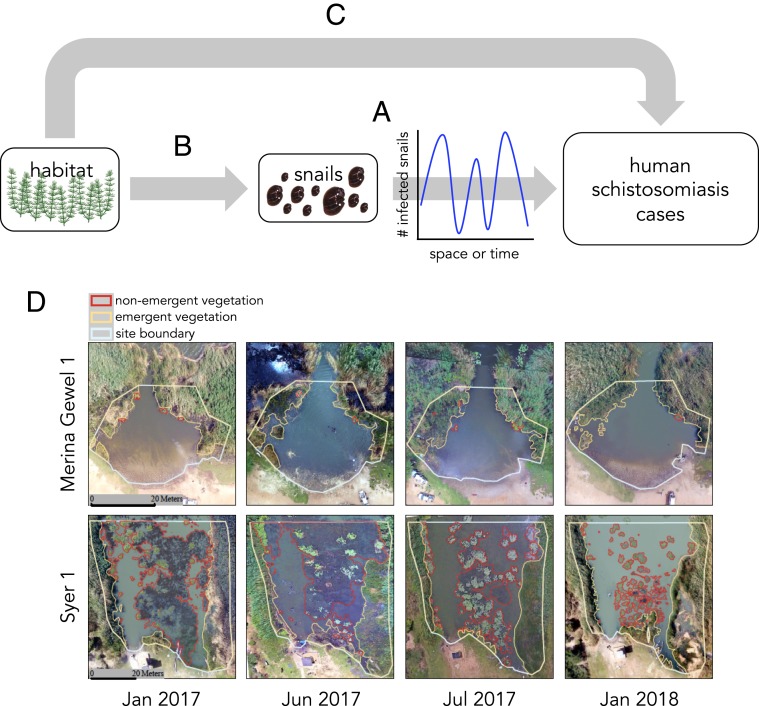
(*A*–*C*) Conceptual diagram. (*A*) Snail density/abundance may not be correlated with human schistosomiasis cases if snails are ephemeral and patchy and therefore difficult to quantify without sampling that is intensive in space and time. (*B*) If the presence of suitable snail habitat is both stable in space and time and an effective predictor of the presence of snails, (*C*) it might provide a better indicator of human schistosomiasis cases than would direct counts of snails. Some environmental predictors of the presence of suitable snail habitat are easily observable by satellite or drones; for example, see (*D*) aerial images taken by drone for representative small-area (Merina Gewel 1) and large-area (Syer 1) sites.

Here, we investigate transmission foci located at the site of the world’s largest recorded schistosomiasis epidemic: the Lower Senegal River Basin in Senegal, above the Diama Dam (25). We were especially interested in a hypothesis first suggested decades ago (18, 19, 25, 26): that snail habitat may be a stable environmental proxy for snails (Fig. 1 *A* and *B*) and could be a more effective predictor of human infection burden than snail counts (Fig. 1*C*). We explored this hypothesis using technology that was not available decades ago: satellite and drone imagery.

We found that schistosomiasis transmission risk was higher near human water contact sites where there was more habitat available for snails. Rather than demanding intensive boots-on-the-ground effort, snail habitat can be observed by drone or satellite, making possible large-scale, fine-grained estimation of human urogenital schistosomiasis risk. The new field of “precision mapping” has focused on mapping human infection burden (27), and our work suggests that habitat mapping might provide an efficient, complementary avenue for identifying transmission foci. In addition to indicating transmission, snail habitat may also drive transmission. Therefore, after identifying high-risk areas, removing the plant species that support snails could reduce the transmission of urogenital schistosomiasis to people, complementing mass drug administration in campaigns to eliminate schistosomiasis.

## Results

Across 32 sites (*SI Appendix*, Fig. S1) and 6 sampling periods spanning 2 y (*SI Appendix*, Fig. S2), we quantified snail densities in 1,922 quadrats, 224 of which contained *Bulinus truncatus/globosus* (*SI Appendix*, Fig. S3). This resulted in records of 3,925 *B. truncatus/globosus* snails, along with site-level data about habitat area (e.g., site size, area of site covered by nonemergent vegetation) for each sampling period (28). Across villages, 76% of enrolled children were infected with urogenital schistosomiasis at the outset of the study, with a geometric-mean egg output of 16.2 eggs per mL of urine. Sixty-four percent were positive in 2017 (i.e., 1 y after receiving praziquantel) and 66% were positive in 2018 (28). Across all statistical models, we omitted significance testing and interpretation of estimated parameters in favor of interpreting the sign and agreement across multiple models for each estimate (29). Similarly, we used simple metrics of predictive power (e.g., mean squared error [MSE]) and posit that variables leading to high predictive power should be beneficial for policy-based interventions against schistosomiasis.

### Identifying Snail-Related Predictors of Human Urogenital Schistosomiasis Burden.

Evidence for a link between snails and per capita reinfection rate varied across statistical models. The model that was top ranked by Bayesian information criterion (BIC) was the null model, suggesting that no snail or snail–habitat variables contributed substantially to explaining the probability of reinfection with *Schistosoma hematobium* (or *S. hematobium–bovis* hybrids). However, the 5 statistical models within 10 ∆BIC of the null model did associate snails with the probability of reinfection with *S. hematobium* (*SI Appendix*, Fig. S4*A* and Tables S1, S2a, and S3). Furthermore, the model with the best predictive power (i.e., lowest MSE) for reinfection rate included a significant positive coefficient for the snail–habitat variable snail abundance (i.e., snail density in each microhabitat * area of each microhabitat; *SI Appendix*, Table S1). The next-best–supported models included terms for the abundance of infected snails (i.e., infected snail density in each microhabitat * area of each microhabitat; a snail–habitat variable), snail density (i.e., snail density weighted by the area of each microhabitat; a snail variable), snail prevalence (i.e., snail prevalence weighted by the area of each microhabitat; a snail variable), and infected snail density (i.e., infected snail density weighted by the area of each microhabitat; a snail–habitat variable; *SI Appendix*, Fig. S4*A* and Tables S2a and S3).

The top 2 models for *S. hematobium* egg counts in infected individuals (i.e., all models within 10 ∆BIC of the top model) both contained snail–habitat variables (*SI Appendix*, Fig. S4*B* and Table S2b). The top model included a positive coefficient for total snail abundance and the second-best model included a positive coefficient for infected snail abundance (*SI Appendix*, Table S4). The models that were top ranked by BIC also had the lowest MSEs (*SI Appendix*, Table S2b), suggesting that these 2 models had the best ability to predict egg counts of all of the models we screened.

### Number, Size, and Persistence of Snail Clusters in Space and Time.

Snails occurred in ephemeral clusters. Across 15 sites (*SI Appendix*, Fig. S3), we identified 30 significant (43 total) clusters of *B. truncatus/globosus* (*SI Appendix*, Fig. S5), which had an average radius of 5.50 m. Including sites where no *B. truncatus/globosus* were found at any time point, there were, on average, only 2.0 clusters per site over 2 y. Among only those sites that contained *B. truncatus/globosus* snails, there were on average 2.5 clusters per site. Although the small number of clusters suggested that snail control could be spatially focused, the locations of most *B. truncatus/globosus* clusters were ephemeral: For 24 of 30 clusters (80.0%), clusters were present during only one sampling period per location within a site. Only 4 clusters (13.3%) persisted across 2 or more time points, and only 2 (6.7%) were detected across 3 consecutive sampling periods (i.e., spanning 1 y), the maximum persistence time that the model is capable of detecting.

**Fig. 2.**
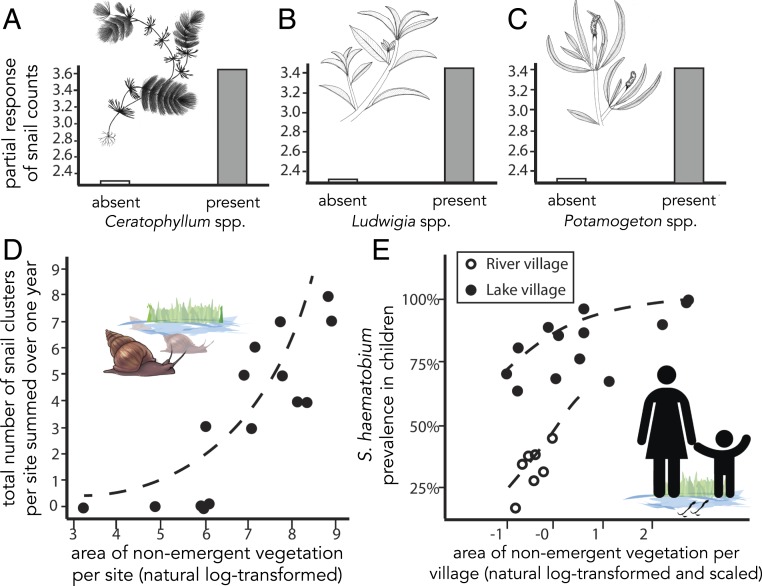
Nonemergent vegetation is associated with human urogenital schistosomiasis risk at quadrat (*A*–*C*), water contact site (*D*), and village (*E*) scales. (*A*–*C*) Partial response of predicted snail count to 3 species of nonemergent vegetation: *Ceratophyllum* spp., *Ludwigia* spp., and *Potamogeton* spp. (*SI Appendix*, Table S6). The partial response of expected snail count (i.e., the product of encounter probability and expected nonzero count) was predicted across the range of each covariate while holding all other covariates at zero, instead of interpreting parameter estimates between model components (as recommended by ref. 65). Images of plant species courtesy of Danielle Claar (University of Washington, Seattle, WA). (*D*) The total number of *B. truncatus/globosus* snail clusters per site increased with area of nonemergent vegetation. The dotted line shows the best-fitting Poisson GLM (*SI Appendix*, Text S6). Points are jittered slightly to aid visualization. (*E*) Prediction plot for males in the top logistic GLMM by BIC. The likelihood of urogenital schistosomiasis infection increased with area of nonemergent vegetation at both river (open circle) and lake (closed circle) villages. Area of nonemergent vegetation per village was calculated as a weighted average among water contact sites within each village, and was natural log-transformed and scaled. Shown are values for villages in each of the 2 y of sampling (2016 to 2018).

New clusters were often present at sites where previous clusters were no longer present. For example, at Ndiawdoune, ephemeral snail clusters (i.e., clusters detected in only one sampling period) were present in 5 of 6 sampling periods, but were never found in the same location (*SI Appendix*, Fig. S5). More clusters occurred at those sites with greater availability of snail habitat (i.e., greater area covered by nonemergent vegetation; Fig. 2*D*).

### Habitat Correlates of Snail Density.

We found several habitat variables that were positively associated with snail density (i.e., counts of snails per quadrat; Fig. 2 *A–C* and *SI Appendix*, Fig. S6 and Table S5). In the best-fitting model, snail density increased with the mass of nonemergent, floating vegetation (*SI Appendix*, Fig. S6*C*) and with the presence of *Ludwigia* spp., *Ceratophyllum* spp., and *Potamogeton* spp. (Fig. 2 *A*–*C* and *SI Appendix*, Fig. S6 *D*–*F* and Tables S6–S9). Snail density per quadrat was higher at river sites than at lake sites (*SI Appendix*, Fig. S6*G* and Table S8). We performed a separate, post hoc analysis and found that—in addition to having more *B. truncatus/globosus* snails per quadrat*—*river sites also had more *B. truncatus/globosus* snails per site (mean estimate, 60,434 snails per site) than did lake sites [mean estimate, 33,232 snails per site; linear regression, *t*_(2,972)_ = −17.97, *P* < 0.0001].

### Identifying Snail- and Habitat-Related Predictors of Human Urogenital Schistosomiasis Burden.

**Fig. 3.**
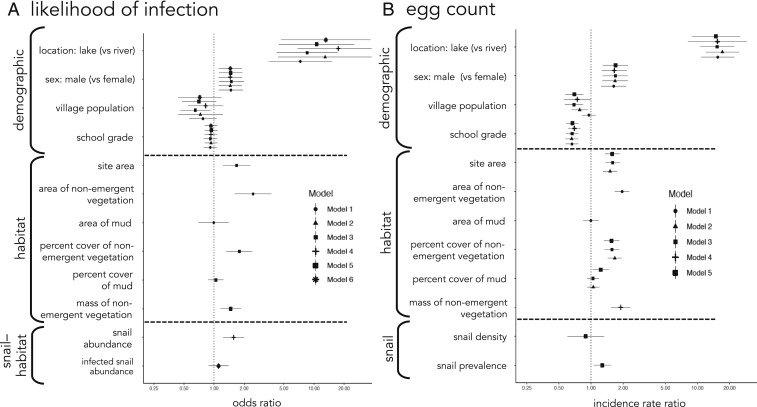
Results of (*A*) logistic GLMM (prevalence) and (*B*) negative binomial GLMM (egg count) aiming to identify habitat-related, snail–habitat, and snail predictors of human urogenital schistosomiasis burden. All models within 10 ∆BIC of the top model are shown here. Models are numbered by their BIC rank and are described in detail in *SI Appendix*, Tables S11 and S12. An odds or incidence rate ratio >1 indicates the predictor is associated with increased risk or burden, and an odds or incidence rate ratio <1 indicates the predictor is associated with decreased risk or infection burden. Error bars indicate 95% confidence intervals.

Models containing habitat variables were better at describing and predicting the probability of reinfection with urogenital schistosomiasis than were models containing only snail variables (Fig. 3*A* and Table 1). Controlling for demographic variables (including age, sex, and village population size, as well as village location in river versus lake habitat), the 6 models that best described human reinfection probability contained the following habitat variables: total area of nonemergent vegetation (i.e., suitable snail habitat), area of water contact site, and percent cover of nonemergent vegetation (*SI Appendix*, Table S10a and Fig. S7*A*). These 6 models cumulatively had 98.9% support by BIC weight (Table 1). The model that was top ranked by both BIC and MSE contained the habitat variables total area of nonemergent vegetation and total area of mud (*SI Appendix*, Table S10a). Some models that contained snail–habitat variables (i.e., total snail abundance at a site, total infected snail abundance at a site) were included among the models within 10 ∆BIC of the top-ranked model. However, these models tended to rank lower by MSE than the models containing habitat variables (Table 1 and *SI Appendix*, Tables S10a and S11), suggesting that the models containing habitat variables had greater ability to predict human reinfection probability than the models containing snail–habitat variables. The top 3 models, which contained no snail-related predictors, cumulatively had 95% support by BIC weight. It is important to note that the only snail-related variables retained in this model selection process were snail–habitat variables (i.e., variables that contained information on both snails and habitat; *SI Appendix*, Table S1).

**Table 1.** Summary of (a) logistic GLMM (individual-level probability that a child became reinfected after praziquantel treatment) and (b) negative binomial GLMM (egg count of reinfected children) aiming to identify habitat-related, snail–habitat, and snail predictors of human urogenital schistosomiasis burden

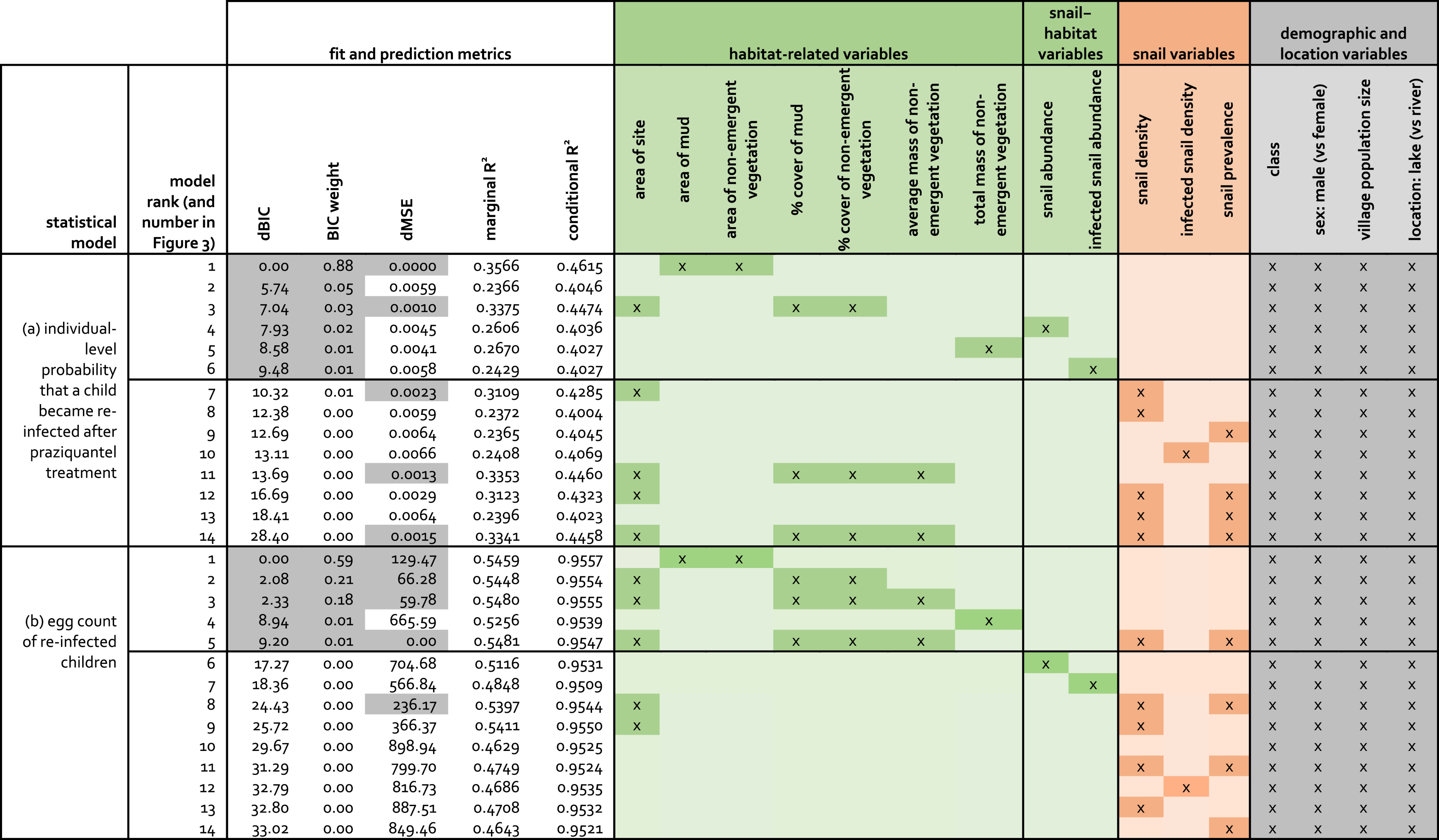

Models are numbered and ordered by their BIC rank. Models within 10 ∆BIC of the top model are marked in gray in the dBIC column. dMSE is indicated, with top models by MSE marked in gray in the dMSE column. Also shown are marginal (i.e., associated with fixed effects) and conditional (i.e., associated with fixed plus random effects) *R*^2^. The remaining columns describe which variables were included in each model. Habitat-related variables contain only information about habitat. Snail variables contain only information about snails. Snail–habitat values are obtained using information about both habitat and snails (*SI Appendix*, Table S1).

Consistent with the top models for probability of human reinfection, models containing habitat variables were better at describing and predicting egg counts in infected individuals than were models containing only snail-related variables (Table 1, Fig. 3*B*, and *SI Appendix*, Table S10b). The models that were top ranked by BIC (i.e., that were within 10 ∆BIC of the top model) contained the following variables: total area of nonemergent vegetation (i.e., suitable snail habitat), area of water contact site, and percent cover of nonemergent vegetation (Fig. 3*B* and *SI Appendix*, Tables S10b and S12 and Fig. S7*B*). These 6 models cumulatively had 99.9% support by BIC weight (Table 1). Egg count was also positively associated with the mass (in grams) of nonemergent vegetation at each village in some of the top-ranking models (Fig. 3*B* and *SI Appendix*, Tables S10b and S12). None of the models containing only snail-related predictors were included among the models that were within 10 ∆BIC of the top model (*SI Appendix*, Table S10b), suggesting that snail variables described little to no variation in egg count. The top 4 models, which contained no snail-related predictors, cumulatively had 99% support by BIC weight. The full orthogonal model was the top-ranked model by MSE, but not by BIC, probably because BIC penalizes for the number of predictors (*SI Appendix*, Table S10b). The full orthogonal model, which contained snail variables, had the highest predictive accuracy of all models (i.e., lowest MSE), but the other 4 models with the next lowest MSEs did not contain any snail or snail–habitat variables (*SI Appendix*, Table S10b).

## Discussion

Our spatially intensive, quantitative snail surveys revealed positive correlations between the distribution of schistosome-competent snails and human infection. However, several easy-to-quantify habitat proxies of snail abundance (e.g., area and percent cover of nonemergent vegetation, site area) were more powerful predictors of human infection. Because snail populations are spatially and temporally variable, any single measurement of their density provides a mere snapshot of risk, whereas actual human burdens integrate risk over both space and time (*SI Appendix*, Fig. S8). The area available for colonization by vegetation used by snails and the area actually covered by these types of vegetation probably integrate this risk, thereby providing a more reliable indicator of human infection burdens (Fig. 1*C*).

Schistosomiasis researchers have often struggled to document local transmission of schistosomiasis (18–24), perhaps because sampling has traditionally been conducted using methods and at resolutions and sampling intensities that are inappropriate for such patchy and dynamic snail populations (Fig. 1). By quantifying snails with intensive sampling and random selection of quadrat locations, we found that integrative snail–habitat variables, like total snail abundance (i.e., snail density in each microhabitat * area of each microhabitat) and total number of infected snails within village water contact sites (i.e., infected snail density in each microhabitat * area of each microhabitat) predicted schistosomiasis reinfection in humans. Although this result seems encouraging, the snail sampling we conducted involved thousands of person-hours of intensive field effort and would be impractical for a snail control campaign.

If snail sampling were to be used to identify villages for schistosomiasis transmission intervention, it would need to be optimized for efficiency. Ideally, it would identify sites within a water body where snails could be efficiently targeted with control efforts (e.g., molluscicide application). We used a cluster analysis to 1) quantify the spatial and temporal variability in snail abundance and 2) quantify the minimum sufficient spatial extent and duration for snail control efforts. Although snails were clustered, these clusters were so ephemeral (*SI Appendix*, Fig. S5) that focused control efforts would need to act quickly and resample frequently. This seems too impractical to be a general solution for snail control.

Fortunately, there is an alternative for identifying high-risk transmission sites that does not require substantial labor. Snails were tightly associated with certain kinds of aquatic vegetation (Fig. 2). In models predicting human reinfection (Fig. 3*A*) and egg counts (Fig. 3*B*), the best nondemographic predictors of human infection burden 1 y after praziquantel treatment were percent cover of the site by nonemergent vegetation and area of the site, or the product of these 2 quantities: the total area of nonemergent vegetation. Even though these easy-to-measure variables are associated only indirectly with the parasite’s life cycle (Fig. 1*C*), they predicted infection risk better than did data from our intensive snail sampling efforts.

Our study specifically took into account the variability in snail abundance and prevalence among microhabitats to estimate village-level snail variables. Previous studies have estimated overall snail density across a water access site; such estimates can be inaccurate if they are not produced by truly random spatial sampling, because snail density varies among microhabitats and the area of these microhabitats varies across sites. By estimating habitat-corrected snail variables, we produced more accurate estimates of actual snail abundance and density at each village, which might explain why we were able to detect associations between snail abundance and human infection burden where previous studies were not. Therefore, even where snail–habitat variables were positive predictors of human infection burden, they still reflect the underlying microhabitat availability, highlighting the importance of quantifying habitat to predict human urogenital schistosomiasis burdens.

The mechanisms linking site size to human schistosomiasis burdens remain to be identified. Site size is independent of snail habitat in our statistical models, but might still contain information regarding snail habitat, because the burden of human schistosomiasis does not merely reflect the abundance of snail habitat at the moment of sampling, but integrates over many months, even years, prior to the moment of sampling. We surmise that the total area cleared of emergent vegetation (i.e., site area) reflects the area available for growth of the plants that serve as snail habitat; the more area, the greater the likelihood that, at some point in the recent past, nonemergent vegetation and snails existed there, and therefore the greater the urogenital schistosomiasis burden in adjacent human communities (Fig. 3). It is also possible that increasing habitat area (i.e., increasing the size of water contact sites) increases rates of snail emigration into the site and, once established, those snails are less likely to go locally extinct (30). People might also choose larger sites for activities that entail elevated exposure to schistosome cercariae (e.g., swimming instead of washing). Larger sites might attract more human activity, perhaps including individuals traveling from outside the local village, thereby intensifying transmission at those sites. Irrespective of human activity levels, larger sites might also have water quality conditions (e.g., lower turbidity, lower temperature) more conducive to cercarial survival and transmissibility. Although we cannot conclusively identify the mechanisms linking site size to human schistosomiasis burden, we suspect that habitat availability for snails plays an important role. Regardless, site size is perhaps the easiest-to-measure site-level variable that predicts human schistosomiasis risk.

Although site size predicts human infection burden, it remains puzzling that site size predicts risk independent of snail density. With increased site size, the distance that cercariae must swim to infect a human bather will increase. Our conclusion that larger sites pose a greater risk of urogenital schistosomiasis transmission because they are more likely to contain snails therefore requires the assumption that cercariae produced by those snails can reach people wading there, either via active swimming in search of hosts, passive movement on currents, or a combination of these. In the course of their short (∼12-h; ref. 31) infectious stages, cercariae can probably move at least a few meters under their own power (32); for example, *Schistosoma mansoni* cercariae added to standing water are able to infect mice held up to 3.1 m away from the point of cercarial release (the greatest distance tested; ref. 33). Compensating for this weak swimming ability, cercariae are chemokinetic and strongly attracted to molecules associated with human skin (34). They also actively swim to the surface (35), where they may be dispersed by currents (33, 36, 37); 10% of experimentally released Schistosoma *japonicum* cercariae were still infective to mice after traveling 100 m downstream at 0.182 m/s (38) and substantial worm burdens were acquired by mice experimentally exposed to *S. mansoni* cercariae even when cercariae were released 97.5 m (39) or 610 m (36) upstream from mice. The water contact sites where our study was based are small enough (average area, 1,607 m^2^) that cercariae produced within these areas could conceivably traverse even the greatest dimension of the largest site (∼121 m), especially considering that cercariae have the capacity to efficiently detect and swim toward human bathers (40). We propose that increased habitat area per site results in more snails per site, and more snails per site could result in more cercariae per site. Even if cercarial density remains the same as site area increases, the ability of cercariae to detect and find a person means that increased area should increase the number of cercariae that manage to infect people.

There were higher snail densities and higher total numbers of snails at river sites relative to lake sites (*SI Appendix*, Fig. S6*G*), but a higher burden of *S. hematobium* among humans living near the lake (Fig. 3). This disconnect could arise for several reasons. It is possible that human behaviors or socioeconomic factors that mediate exposure differ between river and lake villages. River and lake sites might systematically differ in morphology or flow rates in ways that influence schistosomiasis risk.

Senegalese villagers increase their access to water by mechanically clearing and burning emergent vegetation at village shorelines (41). Emergent vegetation like *Typha* spp. and *Phragmites* spp. can expand rapidly into littoral areas if left unchecked, and thereby limit the area of water contact sites and the availability of water to rural villages (42). Our work suggests (but does not prove) that clearing emergent vegetation could exacerbate the transmission of urogenital schistosomiasis by opening habitat for colonization by the nonemergent vegetation types that are associated with high snail density, including *Ceratophyllum* spp., *Ludwigia* spp., and *Potamogeton* spp., and increasing the size of water contact sites—all factors that our analyses suggest might increase the local abundance of snails and, therefore, the risk of urogenital schistosomiasis to humans. Natural freshwaters provide much of the water used by rural people, and some clearance of emergent vegetation is therefore needed to promote human health (i.e., access to water for drinking, bathing, dishwashing, agricultural irrigation, fishing). However, villagers might consider minimizing the amount of vegetation clearing at each water contact site. The relationship between emergent vegetation clearance and net health impact (i.e., the level of emergent vegetation clearance at which an inflection point occurs, where the positive health benefits of water access are overcome by the negative health impacts of elevated schistosomiasis transmission) has not yet been estimated, and additional field studies are needed to assess how changes in the landscape may affect human health.

The results presented here were obtained from observation of sites in northwestern Senegal, and it remains an open question whether our conclusions will apply to other schistosomiasis-endemic regions. We speculate that aerial observation of site size and nonemergent vegetation area could provide effective predictors of human infection burden in other regions, although we caution that this hypothesis remains to be tested. We base our speculation on the fact that schistosome-competent snails have been reported to occur in association with a variety of nonemergent vegetation types across Africa and even in South America (*SI Appendix*, Table S13). We encourage researchers in other regions to use the framework laid out here to assess whether aerial observation and vegetation removal might be useful tools for schistosomiasis surveillance and control, respectively, in study regions outside of Senegal.

Disease control is more efficient when it is focused on the communities that need it. The gold standard for finding high-transmission sites is to collect infection data from humans. However, screening people is time and labor intensive (43–45), and its expense limits the proportion of communities that can be surveyed and resurveyed (46, 47). It is less costly to identify snail habitat, and this can now be done at 2 scales. Previous work has focused on the potential for hyperspectral satellite imagery to correlate land-based vegetation with schistosomiasis transmission (48). Our results suggest that low-cost drone and satellite imagery can be used to map underwater aquatic habitat (i.e., nonemergent vegetation) of *Bulinus* spp. snails, quickly and affordably identifying regional hot spots of risk that can then be targeted with drug administration and environmental control measures. We do not suggest habitat mapping as a replacement for human sampling, but as a complement that can fill gaps in existing epidemiological maps. This intervention would allow a much greater number of villages to be served for the same costs in money and labor currently borne by public health agencies.

Several steps are needed to move from research to application. For public health officials interested in using satellite- or drone-based observation to identify high-transmission villages, we recommend validation of the link between habitat variables and human urogenital schistosomiasis infection burden in the local context and a scale-up step that tests the predictive power of habitat variables for new data, followed by regular monitoring (outlined in detail in *SI Appendix*, Text S1). Because the effort to hand-score aerial images (i.e., drone or satellite images) increases with the spatial extent of the area being scored, it will be time- and labor-intensive to apply this approach at the country level. However, habitat mapping will be even more time- and cost-effective when coupled with recent advances in machine learning, deep learning, and computer vision (49–51), which could automatically produce schistosomiasis risk maps from imagery. Furthermore, because such imagery can be obtained frequently, it should be possible to track how transmission risk changes across seasons and years. Frequent, low-cost habitat mapping could help public health officials stay a step ahead of changes in schistosomiasis transmission, rather than a step behind.

In addition to improving urogenital schistosomiasis surveillance, our results support the suggestion that removing vegetation would kill existing snails and reduce opportunities for snail recolonization. Herbicide and mechanical removal by cutting or use of grazing animals have been suggested as potential interventions (19, 25, 52), and anecdotal evidence suggests that vegetation removal may be effective in small ponds (53) and across small (3-ha) areas of larger lentic ecosystems (54). However, given the ability of many of the implicated plant species to disperse by flotation and regrow rapidly (19, 52), challenges remain for using vegetation removal as a method for snail control. Our study is correlational and thus does not demonstrate a causal relationship between nonemergent vegetation and snail density or between nonemergent vegetation and human urogenital schistosomiasis burden. However, our group is currently performing a controlled field experiment to assess the effectiveness of *Ceratophyllum* spp. removal as an intervention for urogenital schistosomiasis in Senegal. If the experiment demonstrates a reduction in schistosomiasis reinfection with vegetation removal, we could have another tool in our arsenal for managing schistosomiasis, and one that responds to the WHO’s mandate to identify improved approaches for snail control (10, 16, 17).

This study shows that water contact site size and snail habitat area are predictors of human urogenital schistosomiasis burden at a highly endemic schistosomiasis focus, and suggests that this is because increasing the area where emergent vegetation has been cleared increases the likelihood of snails being present and abundant—a fact previously overlooked due to ephemeral patchiness of snails and the small spatial and temporal scales of snail sampling. Because human schistosomiasis burdens reflect risk integrated over space and time, stable indicators of snail presence (e.g., site size, presence of snail habitat) are more effective for predicting risk than are instantaneous snail presence or prevalence. This suggests a few simple interventions that could reduce transmission and urogenital schistosomiasis risk, including limiting water contact site size, removing nonemergent vegetation, and targeting snail control efforts where and when the most snails are predicted to occur.

## Materials and Methods

### Sites.

We selected 32 water contact sites (hereafter, “sites”) distributed across 16 villages in northwestern Senegal (*SI Appendix*, Fig. S1). These 16 villages were chosen to be representative of the rural, high-transmission sites common in the region from among 701 candidate villages (*SI Appendix*, Text S2).

Each of the 32 sites was surveyed 6 times: in May/June 2016, August/September 2016, January 2017, May/June 2017, July/August 2017, and January 2018 (*SI Appendix*, Fig. S2). We chose to sample once per year in each of 3 major seasons: the hot–dry “spring” season, the hot–wet “summer” season, and the cool–dry “winter” season (43). A subset of the 32 sites were involved in parallel manipulative experiments that began in July of 2016; therefore, all analyses exclude data from site–time combinations subject to manipulation (*SI Appendix*, Fig. S3).

### Snail Sampling.

We were interested in assessing the relationship between the presence of schistosome-competent snails and infection burden in nearby humans. Both *B. globosus* and *B. truncatus* were of interest as intermediate hosts of *S. hematobium* (55, 56), causative agent of human urogenital schistosomiasis (*SI Appendix*, Text S3). Patchiness in snail distributions could arise from snails’ strong association with ephemeral environmental features like vegetation (*SI Appendix*, Text S3). Because we wanted to quantify snails as accurately as possible, we made independent measures of their density in open-water/mud-bottom habitat, nonemergent vegetation (e.g., *Ceratophyllum* spp., *Ludwigia* spp., and *Potamogeton* spp.), and emergent vegetation (e.g., *Typha* spp., *Phragmites* spp.) within each site.

We adopted an area-specific technique for snail surveys, which allowed us to explore the spatial and temporal scale of heterogeneity in snail density (details in *SI Appendix*, Text S3). To randomly select snail-sampling locations within each of the sites, we used Google Earth to delineate a boundary around each site (Fig. 1*D*). Fifteen random points were stratified across the 3 microhabitat types (emergent vegetation, nonemergent vegetation, and open water/mud bottom) in proportion to the area of those microhabitats within the boundary of the site. We exhaustively sampled each quadrat (76.2-cm length × 48.26-cm width × 48.26-cm height; area, 0.3677 m^2^), placed all snails found into labeled vials, and returned them to the laboratory, where they were counted, identified to species, measured (shell height to the nearest 0.01 mm), and screened for parasite infection by shedding and dissection. All trematode infections of fork-tailed cercariae (57) were placed individually on WhatmanFTA cards (GE Healthcare Life Sciences) for molecular identification (58) and sequenced, and only snails infected with *S. hematobium* or *S. hematobium–bovis* hybrids were considered to be infected (since these are the only species occurring in *B. truncatus/globosus* that are capable of infecting humans; refs. 59 and 60). Cercariae on FTA cards and snail tissue vouchers were accessioned into the Schistosomiasis Collection at the Natural History Museum (SCAN) (61). Schistosome-competent snails can be sensitive to water conditions, so at each site, we also measured water flow rate, water temperature, salinity, turbidity, pH, and nitrate, nitrite, and phosphate (*SI Appendix*, Text S3).

### Assessing Human Urogenital Schistosomiasis Burden.

After we obtained informed consent from parents/guardians and verbal assent from minor participants, 1,287 school-aged children across 13 villages (12 villages in 2016 to 2017 and 8 villages in 2017 to 2018) participated in this study. At the beginning of the study in 2016, all individuals were screened for schistosomiasis and infected individuals were offered treatment with praziquantel (at 40 mg/kg) to clear infection (*SI Appendix*, Fig. S2). In 2017 and 2018, individuals were again screened and cleared of infection with praziquantel, if needed (*SI Appendix*, Fig. S2). This yielded baseline infection prevalence and egg count estimates in 2016 and reinfection rates 1 y posttreatment in 2017 and 2018 for all participants. All parasite screening was conducted at the Biomedical Research Center Espoir Pour La Santé in Saint-Louis, Senegal, using standardized urine filtration protocols (described in ref. 62). The study received approval from the National Committee of Ethics for Health Research from the Republic of Senegal (protocol no. SEN14/33) as well as the Institutional Review Boards of Stanford University (protocol no. 32196) and the University of California, Santa Barbara (protocol no. 19-170676).

### Identifying Snail-Related Predictors of Human Urogenital Schistosomiasis Burden.

We sought to develop estimates of snail abundance, density, and prevalence that accounted for the variability in these factors across microhabitats within a site. We therefore developed habitat-corrected estimates of each snail-related variable, as described below and in *SI Appendix*, Table S1. While all snail-related variables accounted for variation across microhabitats, we distinguish between “snail variables,” which are variables that contain habitat-corrected snail information (e.g., snail density was the weighted average of snail density across microhabitats, with weight determined by microhabitat area) and “snail–habitat variables,” which contain both snail and habitat information (e.g., snail abundance was the sum of number of snails per microhabitat, with number of snails calculated by multiplying microhabitat-specific snail density by microhabitat area).

To analyze the relationship between snail counts (which were collected at the site level in each field mission) and human infection (which were collected at the village level in each year), we needed to aggregate snail data. This was accomplished by first multiplying the density of *B. truncatus/globosus* snails in each microhabitat type by the area of that microhabitat per site to obtain an estimated number of snails per site. For villages with more than one water contact site, we took a weighted mean of number of snails per site (weighted by site area). Finally, we assumed that risk is cumulative across the year and summed the number of snails per village across all field missions within each year in which reinfection in humans was measured (year 1: May/June 2016, August/September 2016, and January 2017; year 2: May/June 2017, July/August 2017, and January 2018; *SI Appendix*, Table S1).

Using a model comparison approach (63), we investigated whether snail and snail–habitat variables could describe and predict the probability that a child became reinfected after praziquantel treatment (logistic generalized linear mixed model [GLMM] with logit link; function glmer in package *lme4*; ref. 64) and the egg count of infected children (negative binomial GLMM with log link; function glmmTMB from package *glmmTMB*; ref. 65). We compared a suite of snail-related predictors, many of which covaried with one another: the snail variables (i.e., percentage of snails infected with *S. hematobium* or *S. hematobium–S. bovis* hybrids, density of snails, and density of infected snails; *SI Appendix*, Table S1) and the snail–habitat variables (i.e., snail abundance and infected snail abundance). We considered 7 models based on a priori hypotheses about combinations of the predictors that might be predictive, including a full model with the maximum number of predictors that were noncollinear to each other and all possible reduced, noncollinear models. Continuous predictors were centered and scaled by their mean and SD. All models included village population size, whether the village was situated on a river (i.e., the Senegal River or Lampsar River) or on a lake (i.e., Lac de Guiers), individual age and sex, and random effects for individual identity nested within villages and across time. Models were compared based on the BIC (66) to identify all models within 10 ∆BIC of the top model. This cutoff was chosen to be maximally conservative (as recommended by ref. 67). To compare the predictive performance of each model, we conducted leave-one-out cross-validation, calculating MSE for each model. We addressed spatial autocorrelation with a permutation test for Moran’s *I* (*SI Appendix*, Table S2 and Text S4).

### Number, Size, and Persistence of Snail Clusters in Space and Time.

Although heterogeneity in space and time hampers efforts to estimate mean snail density, it might make it possible to focus control efforts efficiently, provided that snail clusters are predictable and persistent. We therefore sought to quantify this heterogeneity with a spatial cluster analysis, which tested the degree to which snails are aggregated in space, and how long snail aggregations persist at a particular location. We performed retrospective space–time scanning with SaTScan, version 9.4.4 (68), to find space–time clusters of *B. truncatus/globosus* snails using discrete space–time permutation models (69, 70). This analysis focused on the 15 sites (distributed across 7 villages) that were unmanipulated throughout the 2-y duration of the study (*SI Appendix*, Fig. S3). The SaTScan algorithm used circular, spatial, moving, varying diameter windows to detect clusters in space and time; clusters occur where there are more cases observed within the scanning window than expected under circumstances of random distribution in space and time (ref. 68 and *SI Appendix*, Text S5). We hypothesized that cluster density would increase with the availability of snail habitat (i.e., area within a site that was covered by nonemergent vegetation) and used a Poisson GLMM with log link to test this hypothesis (*SI Appendix*, Text S6).

### Habitat Correlates of Snail Density.

To identify effective predictors of snail presence/abundance that would be 1) easier to measure and 2) more stable in time and space than snail or snail–habitat variables, we compared a series of delta (i.e., hurdle) Poisson-lognormal GLMMs that related density of *B. truncatus/globosus* to quadrat- and site-level habitat covariates that we identified as potentially important a priori (*SI Appendix*, Table S1 and Text S7). These models included data on all 32 sites (distributed across 16 villages) but excluded site–time combinations that were experimentally manipulated (*SI Appendix*, Fig. S3).

A forward and backward model selection approach based on BIC was used to explore which habitat variables were associated with *B. truncatus/globosus* density (71). This approach was selected because of the reduced computation time relative to an all-subsets selection approach and because forward and backward model selection is an acceptable alternative to all-subsets selection (ref. 72 and *SI Appendix*, Text S7). In total, 23 habitat covariates were considered for model selection.

### Calculating Site-Level Characteristics.

Given that some habitat correlates appeared to be effective predictors of snail abundance in the analysis above, we were interested in evaluating whether habitat variables were better predictors of village-level human schistosomiasis burdens than were snail and snail–habitat variables. Previous studies have identified several species of nonemergent vegetation as appropriate snail habitat (e.g., refs. 25, 26, 52, and 73–78), and suggested that the presence of these species might be an effective predictor of both snail presence (Fig. 1*B*) and human infection burdens (Fig. 1*C*). We therefore characterized the area of each site and the area of each major habitat type—mud, emergent vegetation, and nonemergent (includes floating) vegetation—with a combination of Google Earth imagery (in the first 2 field missions, May/June and August/September 2016) and imagery obtained by an unmanned aerial vehicle (in the final 4 field missions, January 2017, May/June 2017, July/August 2017, and January 2018; 2016 DJI Phantom 4 UAV equipped with an onboard 12.4-megapixel camera; *SI Appendix*, Text S8).

### Identifying Snail- and Habitat-Related Predictors of Human Urogenital Schistosomiasis Burden.

To compare the ability to predict human urogenital schistosomiasis burden across habitat, snail, and snail–habitat variables, we used the same model comparison approach that was used to associate snail and snail–habitat variables with human infection (see above; ref. 63). We sought to identify which village-level factors best described individual-level probability that a child became reinfected after praziquantel treatment [logistic GLMM with logit link; function glmer() in package *lme4*; ref. 64] and the egg count of reinfected children [negative binomial GLMM with log link; function glmmTMB() from package *glmmTMB*; ref. 65]. In addition to the snail variables (i.e., percent of snails infected with *S. hematobium* or *S. hematobium–S. bovis* hybrids, density of snails, and density of infected snails) and the snail–habitat variables (i.e., snail abundance and infected snail abundance; *SI Appendix*, Table S1), we also included habitat variables: percentage of site area covered by nonemergent vegetation (snail habitat), percentage of site area covered by mud, total area (in square meters) of suitable snail habitat (i.e., total area of nonemergent vegetation), and total size (in square meters) of sites (*SI Appendix*, Table S1). We chose a set of models based on a priori hypotheses about combinations of the predictors that might be predictive, including a full model with the maximum number of predictors that were noncollinear to each other and all possible reduced, noncollinear models (including all of the models in the analysis limited to snail and snail–habitat variables, above). This resulted in 14 model variations for each response variable, and we checked the fit of each model by plotting observed versus predicted values for each model (*SI Appendix*, Fig. S7). Models were compared based on BIC (66) to identify all models within 10 ∆BIC of the top model. This cutoff was chosen to be maximally conservative (as recommended by ref. 67). The predictive ability of each model was compared by using leave-one-out cross-validation to calculate MSE for each model. The explanatory ability of each model was assessed by calculating marginal and conditional *R*^*2*^ for each model using the *r.squaredGLMM()* function in the R package MuMIn. To test for spatial autocorrelation among villages in these models, we conducted a permutation test to estimate the Moran’s *I* statistic (*SI Appendix*, Table S10 and Text S4).

## Supplementary Material

Supplementary File
