# Supplementary material for: Precision mapping of snail habitat provides a powerful indicator of human schistosomiasis transmission
Source: Proc Natl Acad Sci U S A. 2019 Oct 28;116(46):23182–91. doi: 10.1073/pnas.1903698116 (PMC6859407; doi:10.1073/pnas.1903698116)
Supplement: Supplementary File [file pnas.1903698116.sapp.pdf]

SUPPORTING INFORMATION

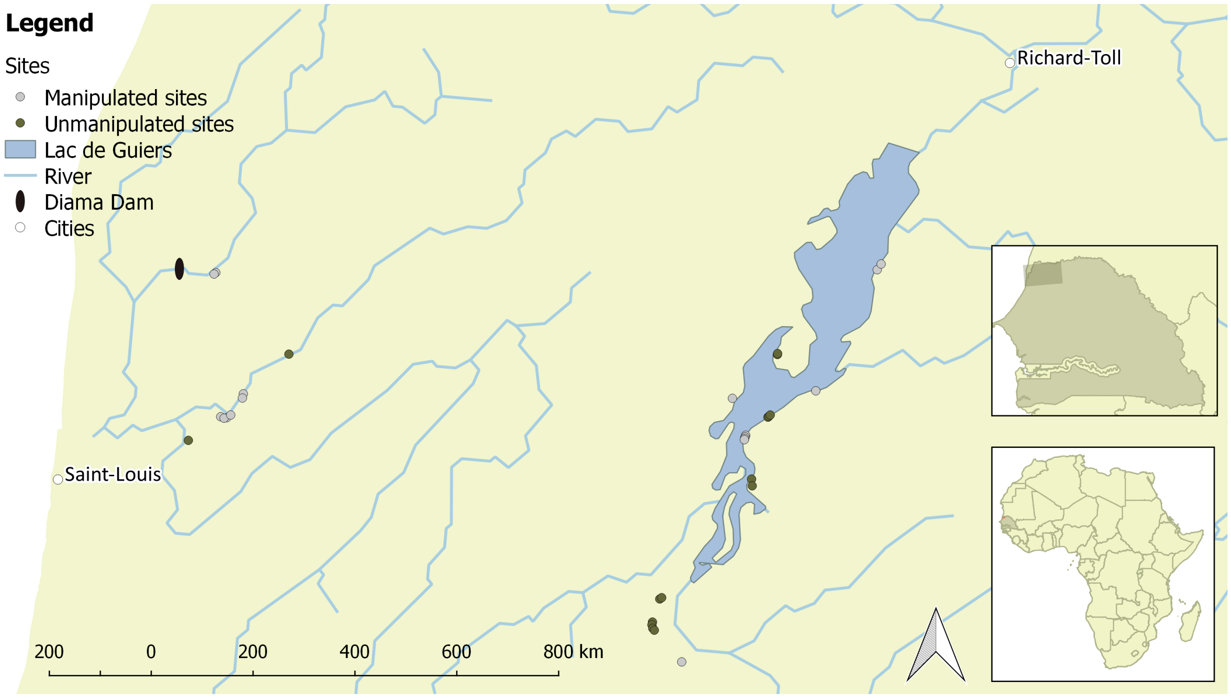

SI Appendix, **Figure S1.** Map of the study area. Dark grey points indicate sites that were unmanipulated throughout the study period, and therefore used in snail cluster and human re-infection analyses. Light grey points indicate sites that were manipulated, where time points prior to manipulation were included in analyses of habitat correlates of snail density. Sites in the western half of the region are located on the Senegal and Lampsar Rivers, while those in the eastern half of the region are located on the Lac de Guiers.

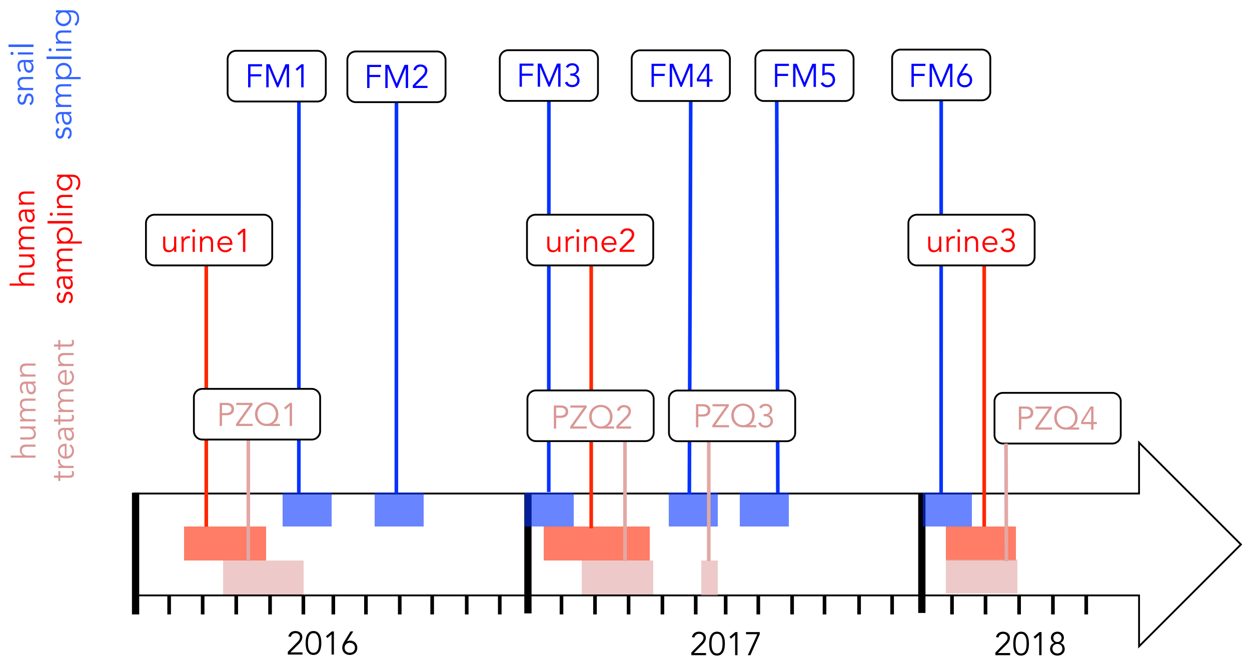

***SI Appendix,* Figure S2.** Timeline for snail sampling, human sampling (i.e., urine collection, filtration, and *Schistosoma* egg counts), and human treatment (i.e., administration of praziquantel to infected individuals). FM = field mission in which snails were quantified, urine = urine collection and filtration, PZQ = praziquantel administration. Tick marks delineate months.

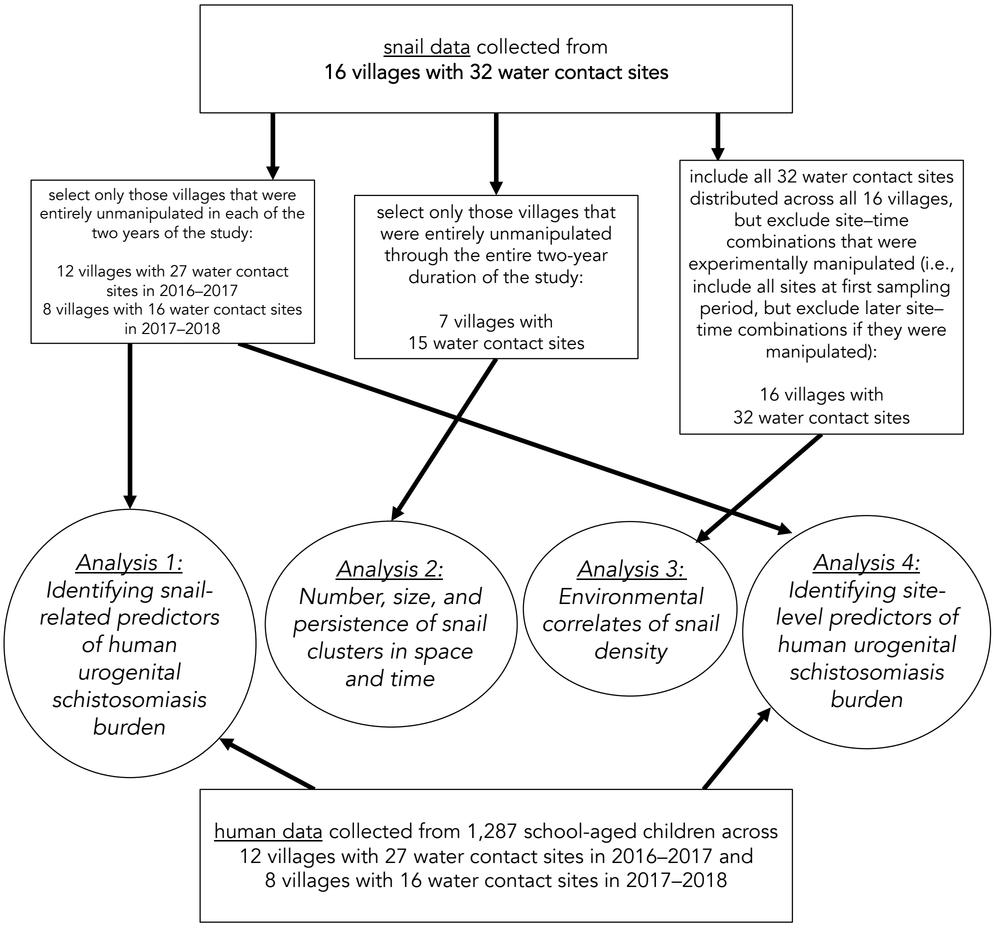

SI Appendix, **Figure S3**. Schematic illustrating the decision-making process for how villages and water contact sites were excluded in each of our four analyses. A subset of the 16 villages originally sampled were involved in two parallel manipulative experiments that began in July of 2016 and June 2017, respectively; therefore, all analyses exclude data from site–time combinations subject to manipulation. However, in order to maximize sample size, we made decisions about which site–time combinations to exclude on a per-analysis basis. This figure describes how those exclusion decisions were made. For Analyses 1 and 4, which investigated the influence of snail, snail–habitat, and habitat variables on human infection burden, we wanted to include all site–year combinations that were unmanipulated; since human infection burdens were measured annually, year was the most temporally resolved unit of time that was possible to use in these two analyses. In year 1 (2016–2017), there were 12 unmanipulated villages (i.e., 4 villages were involved in the manipulative study that began in July of 2016). In year 2 (2017-2018), an additional 2 villages were dedicated to the first manipulative study, and an additional 3 villages were dedicated to the second manipulative study, which began in June of 2017; however, 1 village that had been involved in the first manipulative study (Mbakhana) in year 1 was restored to its natural state by removal of all experimental manipulations in year 2, and we therefore had a total of 8 unmanipulated villages in year 2. For Analysis 2, we aimed to track change over time in the distribution, size, and permanence of snail clusters in the complete absence of manipulation, so we only used those villages that had never experienced any experimental manipulation (i.e., 7 villages). For Analysis 3, we were looking for associations within between habitat variables and snail abundance, and therefore did not need to limit ourselves to sites that went unmanipulated for an entire year or the entire duration of the study. We therefore included the data from the first time point across all sites, since no sites were manipulated in May of 2016. For later time points, we excluded only those site–time combinations that were manipulated.

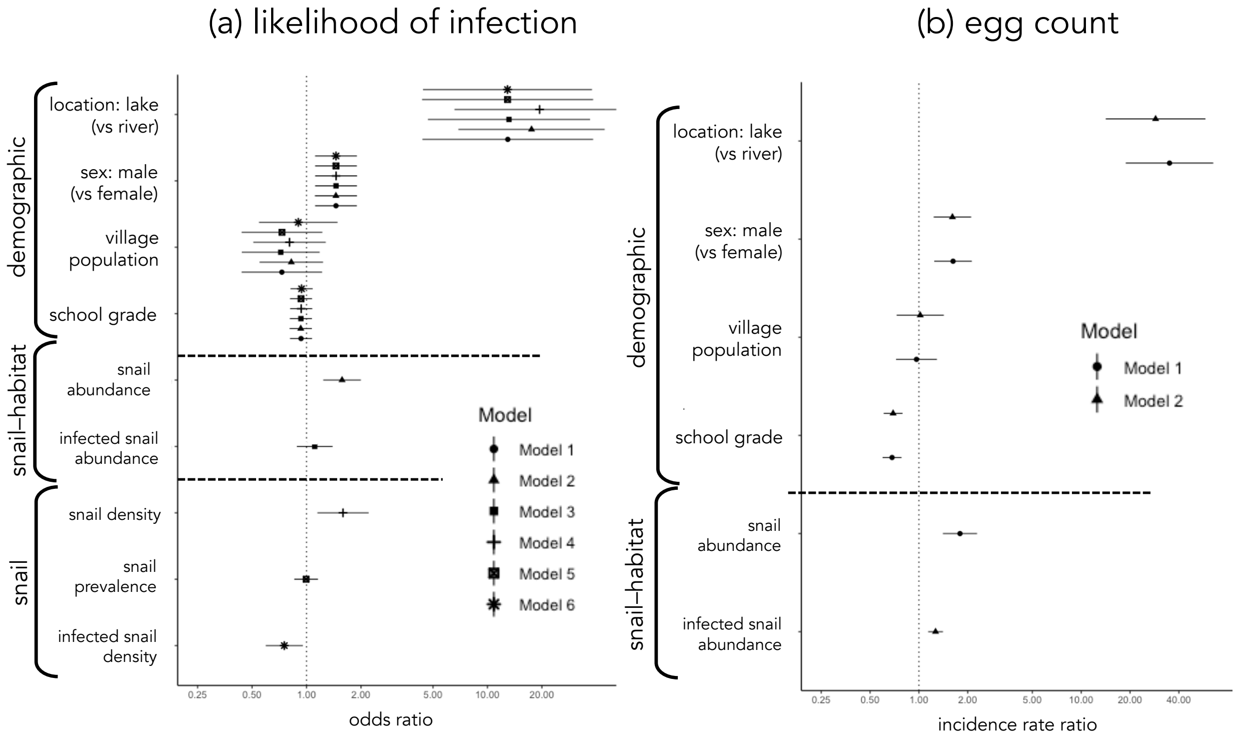

***SI Appendix,* Figure S4.** Results of (a) logistic GLMM (prevalence) and (b) negative binomial GLMM (egg count) identifying snail and snail–habitat predictors of human urogenital schistosomiasis burden. All models within 10 ΔBIC of the top model are shown here; six models for the logistic GLMM and two models for the negative binomial GLMM were within 10 ΔBIC of the top model. Models are described in detail in ***SI Appendix,* Table S2, Table S3,** and **Table S4**. An odds or incidence rate ratio > 1 indicates that the predictor is associated with increased risk or burden, and an odds or incidence rate ratio < 1 indicates that the predictor is associated with decreased risk or burden. Error bars indicate 95% confidence intervals. Demographic predictors are listed at the top of the y-axis, followed by snail–habitat variables, and then snail variables. These results are from models that used only snail and snail–habitat variables (i.e., no habitat variables) to predict human infection burden, and are described in main text under the heading, "*Identifying snail-related predictors of human urogenital schistosomiasis burden*."

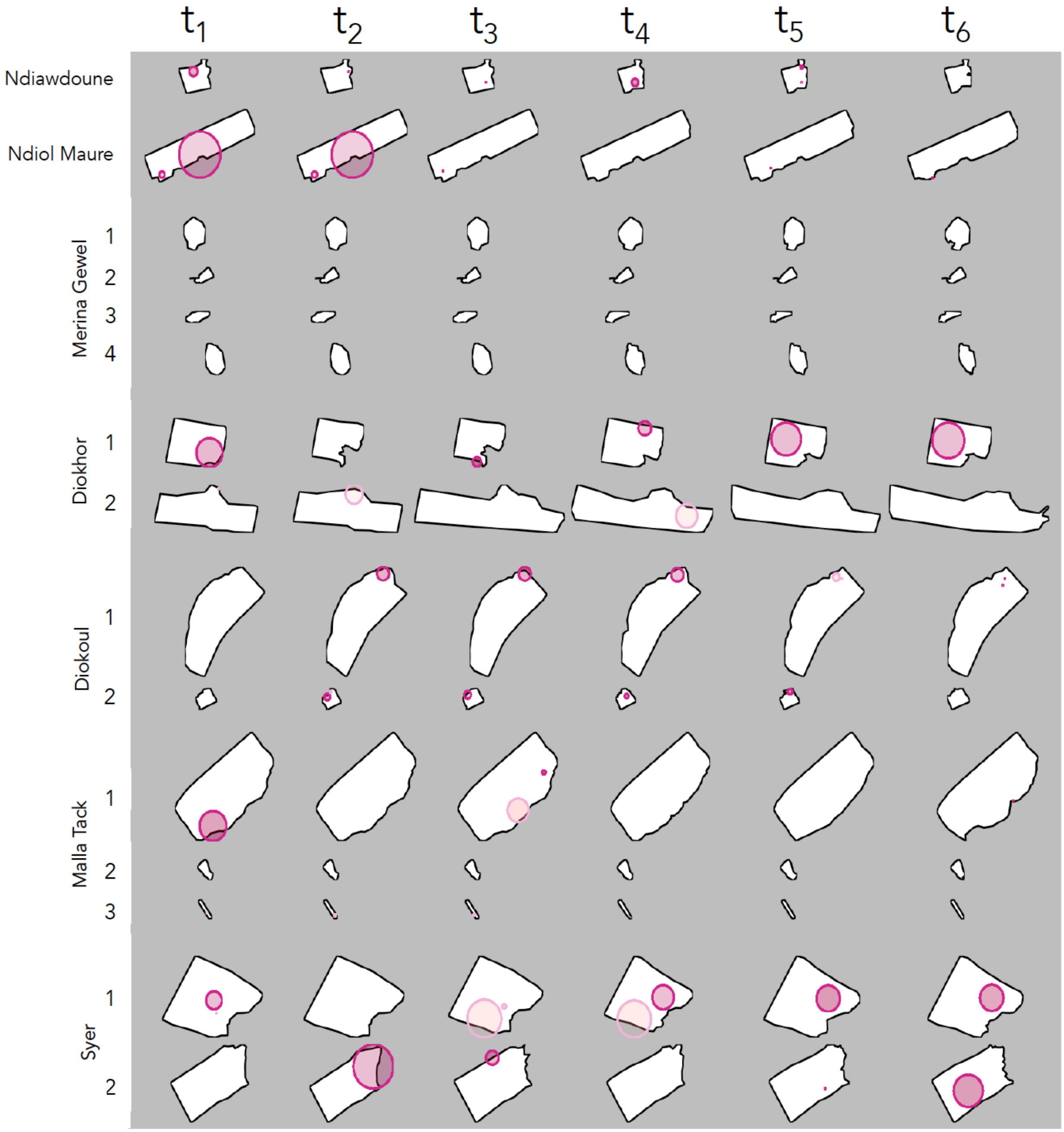

SI Appendix, **Figure S5.** Maps of snail clusters for each site in each of six field missions. Sites are grouped by village and arranged along the y-axis from least (top) to greatest (bottom) total area across sites within a village. Maps are arranged along the x-axis from first sampling period (t_1) to last sampling period (t_6). Dark pink circles indicate significant clusters (at alpha = 0.05), and light pink circles indicate non-significant clusters. Clusters range in size from radius = 0.3421–24 m (area = 0.3677–1810 m^2), and map dimensions are constrained to a fixed 1:1 aspect ratio. Note that site boundaries vary slightly between sampling periods due to temporal fluctuation in water levels and that the size of each cluster represents its spatial extent, not the number or density of snails within that cluster.

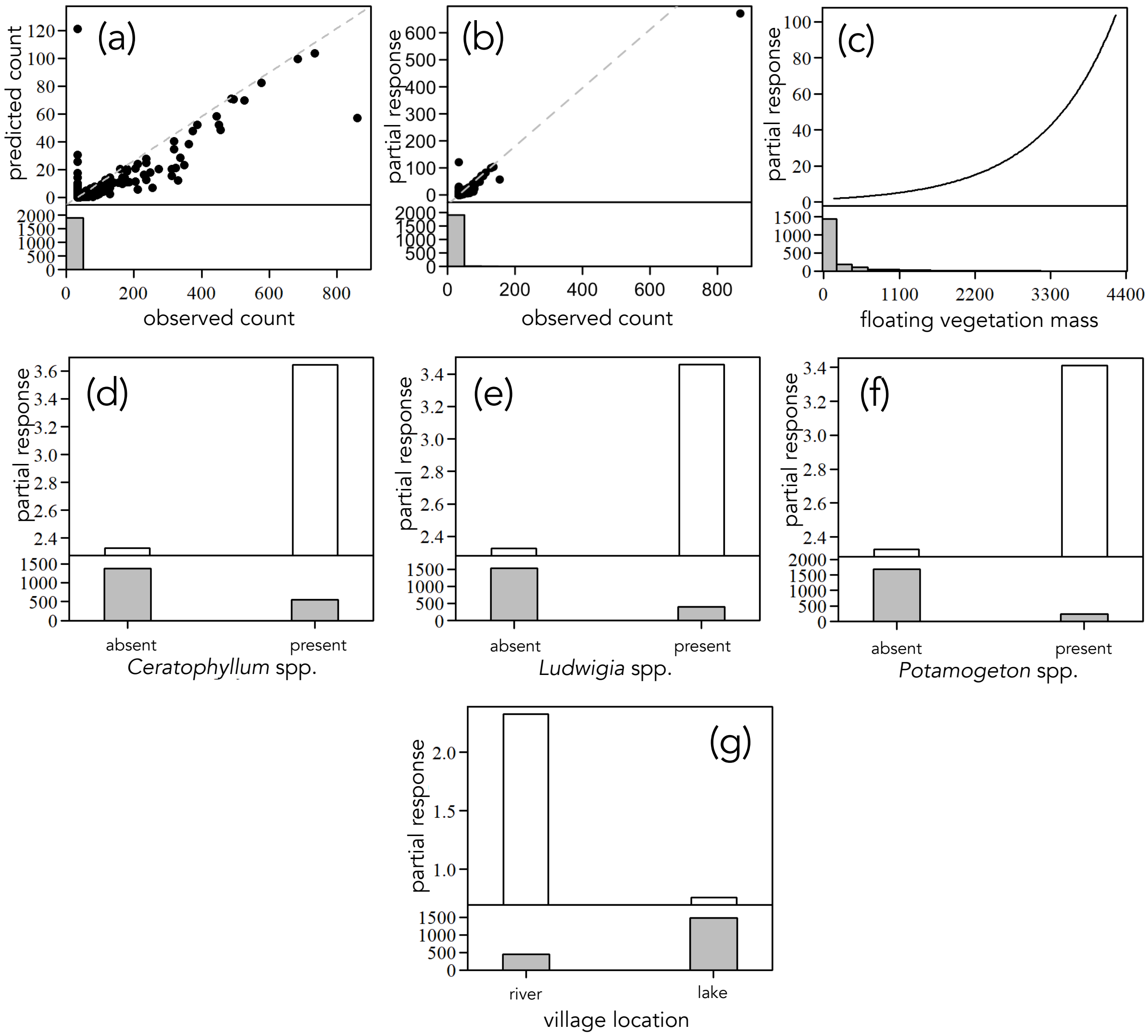

SI Appendix,* Figure S6.** Results from analysis of habitat correlates of snail density. (a) Observed versus predicted counts of *Bulinus truncatus/globosus* from a delta-Poisson-lognormal regression model, with one outlier removed. (b) Observed versus predicted counts of *Bulinus truncatus/globosus* from a delta-Poisson-lognormal regression model, with one outlier retained. (c–g) Partial response of predicted snail counts (i.e., the product of encounter probability and expected non-zero count) was predicted across the range of each covariate while holding all other covariates at zero, instead of interpreting parameter estimates between model components (as recommended by ref. 1). Shown are partial responses of predicted snail count to (c) mass of floating vegetation, (d) the presence of *Ceratophyllum* spp., (e) the presence of *Ludwigia* spp., (f) the presence of *Potamogeton* spp., and (g) whether the village was located on the river or lake (SI Appendix,* Table S6**). Histograms of observations are indicated in grey.

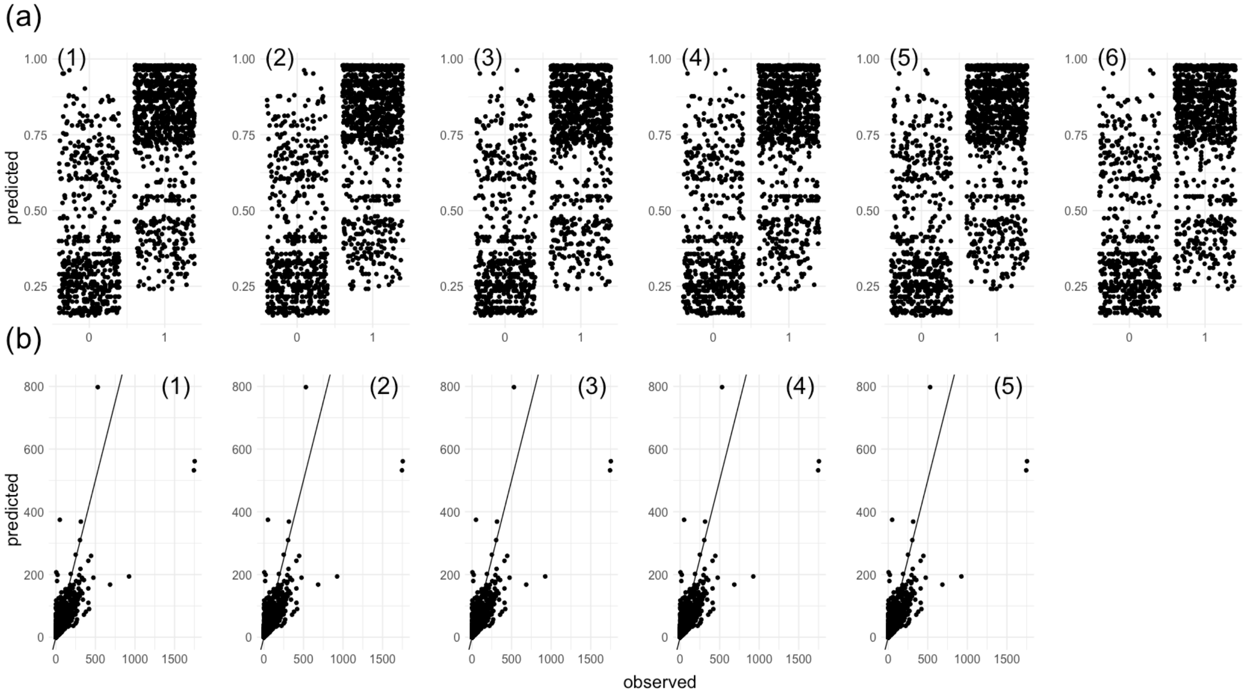

SI Appendix, **Figure S7.** Assessing model fit for the analysis of human infection burden (see "*Identifying snail- and habitat-related predictors of human urogenital schistosomiasis burden*" in main text). We evaluated fourteen alternative model variations for each response variable and here we show observed versus predicted values for those models that are interpreted in the main text (i.e., those within 10 ΔBIC of the top model) for: (a) individual-level probability that a child became re-infected after praziquantel treatment (logistic GLMM with logit link) and (b) the egg count of re-infected children (negative binomial GLMM with log link). Numerals correspond to the models shown in **Table 1a** (for logistic GLMM [individual-level probability that a child became re-infected after praziquantel treatment]) and **Table 1b** negative binomial GLMM (egg count of re-infected children). The effects included in each of the model variations were: (a, 1) area of mud + area of non-emergent vegetation, (a, 2) null model (demographic effects only), (a, 3) area of site + percent cover of mud + percent cover of non-emergent vegetation, (a, 4) snail abundance, (a, 5) total mass of non-emergent vegetation, (a, 6) infected snail abundance, (b, 1) area of mud + area of non-emergent vegetation, (b, 2) area of site + percent cover of mud + percent cover of non-emergent vegetation, (b, 3) area of site + percent cover of mud + percent cover of non-emergent vegetation + average mass of non-emergent vegetation, (b, 4) total mass of non-emergent vegetation, (b, 5) full orthogonal model (area of site + average mass of non-emergent vegetation + snail density + snail prevalence + percent cover of mud + percent cover of non-emergent vegetation).

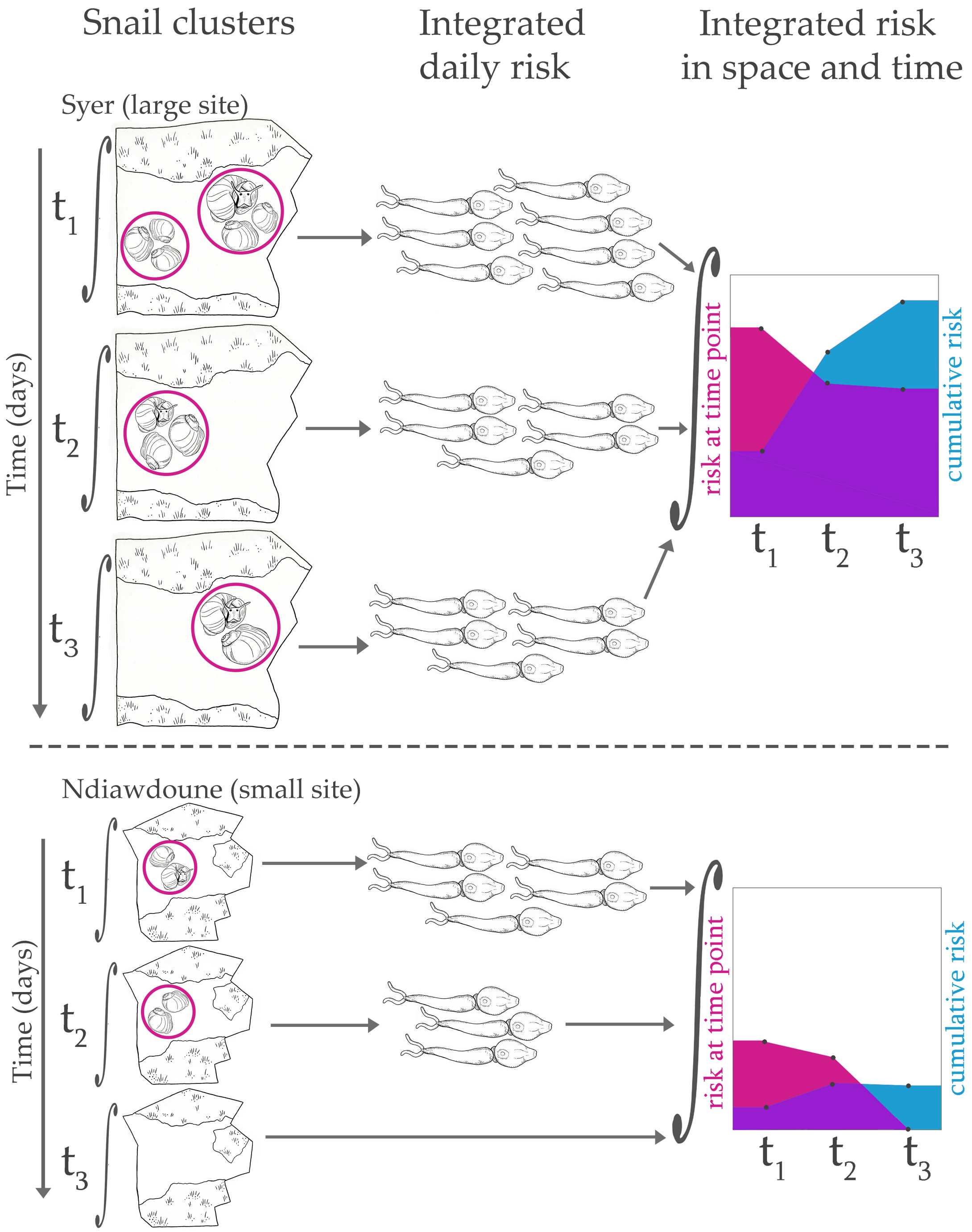

***SI Appendix,* Figure S8.** Conceptual diagram illustrating links between site size (shown are one representative large site, Syer, and one representative small site, Ndiawdoune), ephemerality of snail distributions, risk, and human burden, which integrates across spatial and temporal variability in risk. Time points are indicated as t_1, t_2, and t_3, and represent field missions separated in time by several months. Figure courtesy of Karissa Shutt (artist).

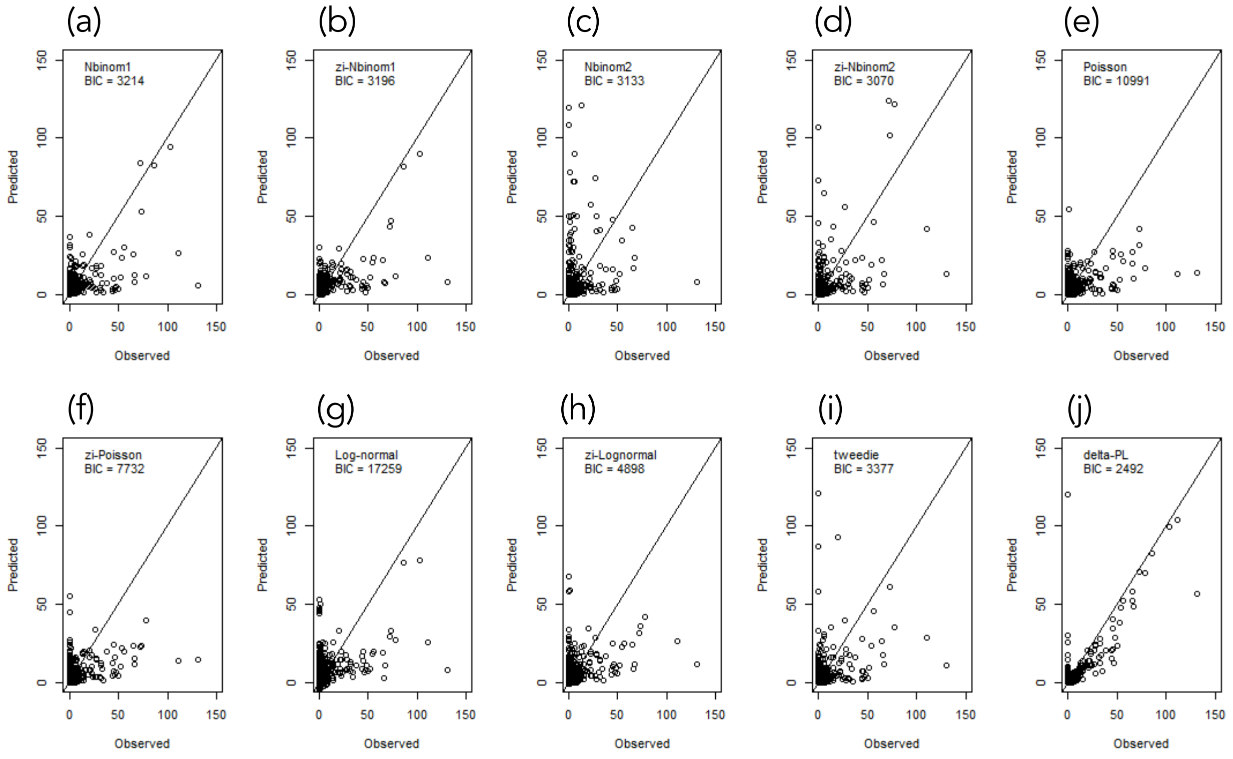

SI Appendix, **Figure S9.** Initial exploration of model formulations for the analysis of habitat correlates of snail density (see "*Habitat correlates of snail density*" in main text and ***SI Appendix,*** **Text S6**, below). We evaluated ten alternative model formulations including: (a) negative binomial where variance increases linearly with the mean, (b) zero-inflated negative binomial where variance increases linearly with the mean, (c) negative binomial where the variance increases quadratically with the mean, (c) zero-inflated negative binomial where the variance increases quadratically with the mean, (e) Poisson, (f) zero-inflated Poisson, (g) lognormal, (h) zero-inflated lognormal, (i) Tweedie, and (j) delta Poisson-lognormal. All models assumed a log link. The model formulation with superior fit was delta-Poisson.

***SI Appendix,* Text S1. Recommendations for public health agencies interested in using satellite- or drone-based observation to identify villages with high rates of urogenital schistosomiasis transmission**

We suggest three steps that public health agencies can take to identify high-transmission villages: (*i*) validation in the local context, (*ii*) scale-up, and (*iii*) regular monitoring. Step 1 will be required in regions outside the region tested here (i.e., northwestern Senegal), to verify that associations between aquatic vegetation, snails, and human schistosomiasis burden hold in each location. Validation should involve making drone- or satellite-based maps (or both) of the extent of non-emergent vegetation, percent cover of non-emergent vegetation, and site size at sites where human infection burdens have been established by urine filtration (i.e., sites known to have both high and low schistosomiasis burdens), and statistically associating habitat metrics with data on human re-infection. The choice of whether to use satellites or drones will depend upon (*i*) whether local species of non-emergent vegetation can be reliably discriminated against other species of vegetation with satellite imagery, (*ii*) the frequency with which no-cost (e.g., Google Earth) satellite images are available, versus the desired frequency, and (*iii*) the resources at hand (e.g., consumer-grade drones cost ~USD$1000, whereas Google Earth is free). By scaling up (step 2) to a larger subset of sites, further studies can determine how generally the selected habitat metric predicts schistosomiasis transmission hotspots. For this purpose, human re-infection data are already available through a recent series of Schistosomiasis Operational Research and Evaluation (SCORE) program studies, which have tested hundreds of thousands of participants and quantified reinfection burdens after praziquantel administration in mass drug administration efforts across half a dozen countries in sub-Saharan Africa (2-8). In step 3, public health agencies would use their validated metric to conduct regular monitoring of the region of interest. The high frequency and high resolution of newer satellite data sources, such as those from PlanetLabs and Digital Globe Foundations (9, 10), would facilitate seasonally relevant, regular monitoring (step 3). As high-resolution satellite

imagery and low-cost drones become more readily available into the future, this approach will become more efficient and cost-effective. The proposed methodology will allow for detection of schistosomiasis transmission hotspots at a greater number of villages across greater spatial extents than is currently possible. It will also allow for more efficient targeting of control efforts, including both drug administration and snail control, so that limited public health dollars can be invested where they are most needed.

***SI Appendix,* Text S2. Site selection**

This study was conducted in 16 villages in northern Senegal, above the Diama Dam in the immediate vicinity of the Senegal River, Lac de Guiers, and connected canals. The selection process was based on a list of 696 villages fewer than 10 km from any water resource, obtained from l'Agence Régionale de Dévelopement, Senegal. Small adjustments to this list reduced the number to 693 villages; "Maka Peul" was listed twice, "Thilla" was merged with "Thilla1" and "Mbodiene" was merged with "Diagamba Mbodiene", as these villages are close to each other and the children go to the same school. The list was supplemented with 8 villages encountered in the field that were not included in the base list, leading to a total of 701 villages. The selection process involved three steps: (*i*) geographical exclusion, (*ii*) preliminary Google Earth evaluation, and (*iii*) field visits.

Villages were selected based on the presence of *Schistosoma* infections and a minimum of one and maximum of four regularly used water contact sites along the lake, river, or irrigation canal near the village, so that water contact for the village was local and predictable. The selected water contact sites were permanent freshwater (not brackish) bodies, in place for a minimum of six months prior to the beginning of our study, and had no recent disturbance (e.g., digging).

Villages were considered for selection if they shared a common lifestyle pattern and represented high-risk sites for schistosomiasis transmission. To achieve this, we excluded large- (top 10th percentile of the 701 villages discussed above, considered urban) and small- (bottom 10th percentile of the 701 villages discussed above, considered rural) sized villages.

Village selection breakdown

1. The study region was geographically limited to the administrative regions immediately touching the Senegal River, Lac de Guiers, or connected irrigation canals between the

Atlantic Ocean and the Lac de Guiers. This included the districts (and respective communes) of St. Louis (Fass Ngom, Gandon), Dagana (Bokhol, Dagana, Diama, Mbane, Richard Toll), and Louga (K. Momar Sarr, Nguer Malal, Syer). Excluded villages were from the district Podor and the communes (in districts): Ndiebene Gandiole (Saint Louis), Gae (Dagana), Ross Bethio (Dagana), and Rosso Senegal (Dagana). This exclusion step removed 301 villages from the base list, reducing it to 400 villages.

2. The remaining villages were viewed on Google Earth (Google Earth 7.1.2.2041) and evaluated based on the presence and/or number of freshwater contact sites that could be seen from the satellite photos. We selected those villages with more than zero and fewer than four water contact sites, as well as those for which the water contact was nearby but the number of contact sites was not discernible from satellite photos and needed to be ground-verified. Only 80% of the original villages identified in the census had information on population size throughout the Lower and Middle Senegal River Basin, so those without population data were excluded and those with population size >125 and <2215 total residents were further evaluated. In summary, based on satellite photos and population size, a further 270 villages were excluded based on: the absence of any nearby, identifiable water contact sites (113 villages in the desert areas), only brackish water contact sites (21 villages), only temporary water contact sites (38 villages), village not found on the map (45 villages), population too small (6 villages), population too large (9 villages), villages merged based on their close proximity (7 villages), villages within a protected area (2 villages), and too much littoral vegetation preventing access to the water contact site (8 villages). A total of 133 villages remained eligible after this first-tier selection procedure.
3. The remaining 133 villages were visited (January 2015 – January 2016) so that we could determine (*i*) the presence and/or number of freshwater contact sites, (*ii*) the presence of a school with a sufficiently large number of students in grades 1–3 (>30 students), (*iii*)

the prevalence of schistosomiasis in schoolchildren at the village, and (*iv*) accessibility of the village during the rainy season. A further 117 villages were excluded based on: the absence of a freshwater contact site close to the village (18 villages), more than 4 water contact site (59 villages), no school in the village (15 villages) or not enough students in the school (10 villages), absence of schistosomiasis in the schoolchildren (2 villages), temporary or only small water contact sites in the village (12 villages), water contact sites on private land (4 villages), location of water contact sites in a protected area (2 villages), villages merged based on their close proximity (3 villages), villages with new and recently dug water contact sites (4 villages), a non-collaborative village chief (1 village), involvement in an outside project (1 village), and non-accessibility in the rainy season (2 villages). This resulted in the selection of 16 villages.

***SI Appendix,* Text S3. Detailed methods: snail sampling**

We were interested in assessing the relationship between the presence of schistosome-competent snails and infection burden in nearby humans. To randomly select snail-sampling locations within each of the sites, we used Google Earth to delineate a boundary around each site (**Figure 1d, main text**). The boundary was 2 m inside emergent vegetation (i.e., *Typha* or *Phragmites* spp.), if present. If no emergent vegetation was present, the boundary bracketed the entire extent of the shoreline used by humans. Due to changes in the water level of the river and lake, site boundaries changed slightly from one visit to the next. In all cases, the off-shore edge of the boundary encompassed the site to ~100 cm depth (i.e., maximum depth safely accessible by technicians wearing waders).

Both *Bulinus globosus* and *B. truncatus* were of interest as intermediate hosts of *Schistosoma haematobium* (11, 12), causative agent of human urogenital schistosomiasis. These snail species also host hybrids of *Schistosoma haematobium x Schistosoma bovis*, which are infectious to humans (13). Given their similar contributions to schistosomiasis transmission and the substantial morphological overlap between these two snail species (14), we did not attempt to distinguish *B. globosus* from *B. truncatus,* and from here forward refer to them as *B. globosus/truncatus.*

Patchiness in snail distributions could arise from snails' strong association with ephemeral habitat features like vegetation. *Bulinus* spp. snails use aquatic vegetation as a habitat refuge, a source of oxygen, and as a surface from which food resources (e.g., diatoms, detritus, algae, bacteria) can be scraped (15). Plant taxa implicated in increasing the abundance of medically important snails include the unrooted, non-emergent, floating genera *Ceratophyllum* (16-22), *Nymphaea* (16, 18, 23, 24), *Pistia* (19, 20), and *Salvinia* (16, 19, 20). The presence and location of non-emergent vegetation within a site can vary dramatically across time (**Figure 1D, main**

text). Because we wanted to quantify snails as accurately as possible, we made independent measures of their density in open-water/mud-bottom habitat, non-emergent vegetation (e.g., the floating, non-emergent species mentioned above), and emergent vegetation (e.g., *Typha, Phragmites*) within each site.

Although snail sampling techniques among studies conducted in Asia (e.g., 25) often control for area, estimates of African snails tend to be conducted with techniques that are not area-specific (e.g., timed searches, sweep nets); this compromises the ability to obtain standardized and comparable estimates of density. We adopted an area-specific technique for snail surveys, which allowed us to explore the spatial and temporal scale of heterogeneity in snail density. Fifteen random points were stratified across three microhabitat types (emergent vegetation, non-emergent vegetation, and open water/mud bottom) in proportion to the area of those microhabitats within the boundary of the site. Upon arriving at each site, two observers visually estimated the percent cover by three microhabitats (emergent vegetation [*Typha* and *Phragmitesmites*], non-emergent vegetation [*Ceratophyllum*, *Potamogeton*, *Ludwigia, Nymphaea,* and *Cyperus*], and open water/mud) within the boundaries of each site. We used on-the-ground estimates rather than satellite estimates of percent cover because the distribution of vegetation at these sites is dynamic (**Figure 1D, main text**), and Google Earth satellite images were often taken months before our visits. We averaged the estimates of the two observers and multiplied this by 15 to obtain a total number of replicate quadrats to be sampled for each microhabitat type in each visit. A few sites were too small to accommodate 15 quadrats while maintaining a minimum distance between points of 0.5 m; at these sites, no fewer than 10 quadrats were sampled.

Our goal was to count all snails in each quadrat so that we would arrive at quantitative estimates of the density of snails per square meter of habitat. Within the sampling boundary of

each site, we generated 60 random sampling points no less than 0.5 m apart from one another with the *spsample()* function of the *sp* package in R and loaded these points into a Trimble R1 high-accuracy GPS using Trimble InSphere software (version 2.01). Each sampling point was located using the Trimble R1 GPS, and visited in the randomly selected sequence; we were careful not to disturb other points as we progressed around the site. Inappropriate points (e.g., points that we discovered by attempting to approach were too deep for safe access) were discarded and appropriate points were sampled until the field team had completed its quota of quadrats for each microhabitat type at that site. At each sampling point, we placed a three-sided, aluminum snail enclosure (76.2 cm length x 48.26 cm width x 48.26 cm height; area = 0.3677 m^2) to contain all material within the quadrat boundaries, encompassing all material in the quadrat from the benthos to the water's surface. We immediately recorded the identity of all plant species and the number of emergent plant stems and measured depth to the nearest cm. We then used a triangular scoop (2.5-mm mesh size) to remove all vegetation and other material (including garbage) contained inside the enclosure into a large tub. The contents of the tub were rinsed with freshwater in a 2.5-mm sieve and carefully examined for attached snails. Floating (i.e., unrooted, non-emergent) vegetation was shaken dry and collectively weighed to the nearest 10 grams. After all vegetation and debris had been examined, we passed at least three scoops of mud from the quadrat through 2.5-mm sieves and examined sieves for snails. To ensure that we examined all quadrats exhaustively, scooping was continued until three consecutive scoops yielded zero snails. All snails were placed into labeled vials and returned to the lab, where they were counted, identified to species, measured (shell height to the nearest 0.01 mm), and screened for parasite infection by shedding and dissection. All trematode infections of fork-tailed cercariae were placed individually on WhatmanFTA© cards (26). DNA was eluted, amplified, and sequenced to distinguish between *Schistosoma haematobium* and *S. haematobium–bovis* hybrids from *S. bovis* and non-schistosome furcocercous trematode species (27). The identification was based on multi-locus analyses with one mitochondrial

(*cox*1) and two nuclear (*ITS*1+2 and 18S) genes. Only snails infected with *S. haematobium* or *S. haematobium–bovis* hybrids were considered to be infected (since these are the only species occurring in *B. truncatus/globosus* that are capable of infecting humans; 13, 28). Cercariae on FTA cards were accessioned into the Schistosomiasis Collection at the Natural History Museum (SCAN; 29).

All snails were identified to species using morphological characteristics and preserved in 95% ethanol and, for a subset, their DNA was sequenced to confirm species identity. Total genomic DNA was isolated from a small amount of snail tissue using the DNeasy Blood and Tissue kit (Qiagen, UK) according to manufacturer's instructions. Amplification of a partial cytochrome oxidase 1 (*cox1*) sequence was carried out on snail vouchers (30). PCR investigations and sequencing conditions were chosen as previously outlined (14). Sequencing was performed on an Applied Biosystems 3730XL analyser (Life Technologies, UK).

Schistosome-competent snails can be sensitive to water conditions, so at each site, we measured these characteristics at the site center, including water flow rate (in m/s, estimated from nearest rotation per minute of a digital Geopacks flowmeter), water temperature (to the nearest ºC), salinity (to the nearest 0.1% Brix on a Grainger Brix refractometer, model #REF112ATC), turbidity (Secchi depth to the nearest cm), pH (to the nearest 0.01 pH unit on a YSI 9500 photometer), and nitrate, nitrite, and phosphate (to the nearest 0.01 mg/L on a YSI 9500 photometer).

***SI Appendix,* Text S4. Detailed methods: addressing spatial autocorrelation in analyses of human infection burden**

We were cognizant of the possibility that any patterns we uncovered could be driven by spatial autocorrelation at the village level; that is, if adjacent or nearby villages are more similar to one another than would be expected by chance, this non-independence could bias results and create artefactual relationships that we might erroneously interpret as legitimate conclusions. To test for spatial autocorrelation among villages in the models described above, we conducted a permutation test to estimate the Moran's *I* statistic. We calculated 999 permutations of the village-level residuals from each model and used a spatial weighting scheme based on inverse distance among villages. We then established the rank of the observed statistic in relation to the 999 simulated values, and estimated a pseudo p-value to test the likelihood that the actual Moran's *I* value diverged from the 999 values generated from random spatial distributions. When we performed this permutation test, we discovered that there was mild but significant positive spatial autocorrelation in the residuals for the logistic models (i.e., models of re-infection probability) and mild but non-significant positive spatial autocorrelation in the residuals for the negative binomial models (i.e., models of egg count). To address this issue, we began by looking at the pairwise distances among villages, and grouping nearby villages together, starting with the nearest villages. We only had to do this once (with our closest pair of villages, Mbakhana and Mbarigot, ~0.63 km apart) to eliminate our spatial autocorrelation issue. Results of the Moran's *I* permutation test for the final models (i.e., models where Mbakhana and Mbarigot are combined into a single village) are shown ***SI Appendix,* Table S2** and ***SI Appendix,* Table S10**. This intervention had minimal quantitative and no qualitative influence on our conclusions. Given the lack of spatial autocorrelation in the residuals of our models, we conclude that our results are not pseudoreplicated and that the patterns we observed are unlikely to be due to Type I error driven by spatial autocorrelation.

***SI Appendix,* Text S5. Detailed methods: estimating the number, size, and persistence of snail clusters in space and time**

Although heterogeneity in space and time hampers efforts to estimate mean snail density, it might make it possible to focus control efforts efficiently, provided that snail clusters are predictable and persistent. We therefore sought to quantify this heterogeneity with a spatial cluster analysis, which would test the degree to which snails are aggregated in space, and how long snail aggregations persist at a particular location. We performed retrospective space–time scanning with SaTScan v.9.4.4 (31) to find space–time clusters of *Bulinus truncatus/globosus* snails using discrete space–time permutation models (32, 33). This analysis focused on the 15 sites (distributed across 10 villages) that were unmanipulated throughout the two-year duration of the study (***SI Appendix,* Figure S2**). The SaTScan algorithm used circular, spatial, moving, varying diameter windows to detect clusters in space and time. Clusters occur where there are more cases observed within the scanning window than expected under circumstances of random distribution in space and time (32). Spatial clusters were defined as containing up to 50% of the sampled snails, since a large scanning window (i.e., a large window within which the number of snails observed at each quadrat was compared to the number of snails expected under circumstances of random distribution in space and time) is more likely to contain true clusters and provides good model performance in terms of power, sensitivity, positive predictive value, and misclassification (34, 35). We assumed that the area of the smallest possible patch (one quadrat) would be equal to the area of the quadrat (0.3677 m^2). Temporal clusters were defined by temporal windows containing up to 50% of the study period (i.e., 12 months), the maximum permitted by SatScan software, to maximize power to detect clusters (32, 36). Only non-overlapping clusters were reported, because inclusion of overlapping secondary clusters does not, in general, improve model performance (34). When evaluating the null hypothesis that snails are randomly distributed across space, statistical significance of detected clusters was

assessed using the log-likelihood ratio statistic and the corresponding p-value was obtained using 999 Monte Carlo simulations.

***SI Appendix,* Text S6. Detailed methods: cluster density analysis**

Each spatiotemporal snail cluster identified by our SaTScan analysis represents a potential hotspot of high risk for human infection, and more clusters per sampled area (i.e., cluster density) should indicate higher risk sites. We hypothesized that cluster density and thus disease risk would increase with the availability of snail habitat, which we quantified using the total area within a site that was covered by non-emergent vegetation. We used a Poisson generalized linear model with log link to determine how area of non-emergent vegetation affected the total number of clusters detected per site when summing across sampling periods. Though sampling effort was generally consistent across sites, there was some variability in the number of sampled quadrats due to logistical constraints (i.e., some sites were too small to accommodate 15 quadrats), and we accounted for this variation by including the log number of quadrats sampled per site as an offset in each model. We also natural-log transformed area of non-emergent vegetation so that the few large area estimates would not unduly influence the outcome of our analyses. All analyses were performed in R version 3.5.1, package *MASS* (37), and we compared the two models using the Bayesian information criterion (BIC). Plots of residuals and predictions confirmed that this approach was appropriate for our data. Furthermore, we confirmed that combining cluster data across field missions was appropriate because (*i*) the SaTScan analysis revealed that few clusters persisted across time (see *Results* in main text), (*ii*) a time-explicit regression revealed no temporal autocorrelation in the relationship between clusters and area of non-emergent vegetation, and (*iii*) summing clusters across sampling periods gave the same qualitative results as did analyzing the number of clusters per site per sampling period.

***SI Appendix,* Text S7. Detailed methods: habitat correlates of snail density**

Statistical modelling

To evaluate the relationship between density (count per quadrat) of *Bulinus truncatus/globosus* (host of *Schistosoma haematobium* and *S. haematobium* x *S. bovis* hybrid) and habitat variables, a series of delta Poisson-lognormal models were developed. In the delta model (also known as hurdle model), two distributions are assumed to account for a large number of zero counts: a Bernoulli distribution for the probability of zero counts (*p*) and a Poisson-lognormal distribution for the probability mass of non-zero counts (*c*). The probability function was:

$$f\left(c_i \mid \beta_{0,p}, \beta_{0,c}, \beta_p, \beta_c, \mathbf{x}_{\boldsymbol{i}}, \sigma_{s,p}^2, \sigma_{s,c}^2, \sigma_c^2\right) =$$

$$\begin{cases} p_i & \text{for } c_i = 0 \\ (1-p_i) * \text{Poisson}(c_i \mid \mu_i) & \text{for } c_i > 0 \end{cases}$$

$$\text{logit}(p_i) = \beta_{0,p} + \mathbf{x}_{\boldsymbol{i}}\beta_p + \varepsilon_{s,p}$$

$$\log(\mu_i) = \beta_{0,c} + \mathbf{x}_{\boldsymbol{i}}\beta_c + \delta_i + \varepsilon_{s,c}$$

$$\varepsilon_{s,p} \sim Normal(0, \sigma_{s,p}^2)$$

$$\varepsilon_{s,c} \sim Normal(0, \sigma_{s,c}^2)$$

$$\delta_i \sim Normal(0, \sigma_c^2)$$

where p_i is the probability of zero snails and μ_i is the expected non-zero count of snails from the *i*th quadrat, which are assumed to be a logit- or log-linear function of their respective intercepts ($\beta_{0,p}$ and $\beta_{0,c}$), k habitat covariates ($\mathbf{x}_{\boldsymbol{i}}$), and respective k number of regression coefficients (β_p and β_c). To account for potential overdispersion, a lognormal-Poisson distribution was assumed for the non-zero count data, where δ_i is the deviation for each observation away from the predicted count for its quadrat, assumed to come from a normal distribution with variance σ_c^2. $\varepsilon_{s,p}$ and $\varepsilon_{s,c}$ are random effects to account for site-specific variation around the average zero-probability and expected non-zero count, respectively, assumed to come from normal

distributions with variance $\sigma_{s,p}^2$ and $\sigma_{s,c}^2$, respectively. Expected count ($\bar{c_i}$) for each quadrat was then the product of non-zero expected count μ_i and non-zero encounter probability $(1 - p_i)$. Initial models incorporated within-site spatial and spatio-temporal random effects following methods by (38). However, these were removed due to a lack of convergence and because final models had no residual spatial auto-correlation. Initial efforts evaluated alternative model formulations including: zero-inflated Poisson-lognormal, delta-lognormal, zero-inflated lognormal, delta-Poisson, zero-inflated Poisson, negative binomial, and zero-inflated binomial. However, these formulations provided qualitatively inferior fits to the data (***SI Appendix,*** **Figure S9**).

Model selection and missing data

A forward and backward model selection approach via Bayesian Information Criterion (BIC) was used to explore which habitat covariates were associated with counts of *B. truncatus/globosus*. Starting from an intercept-only model, covariates were sequentially added if inclusion led to reduced BIC. Included covariates were then sequentially removed if removal led to reduced BIC. A covariate was not included if convergence was not achieved upon inclusion. *k* = 23 habitat covariates were considered for model selection (***SI Appendix,*** **Table S5**), based on the hypotheses in ***SI Appendix,*** **Table S1**. All continuous covariates were scaled by their mean and standard deviation prior to analysis. BIC was used in place of Akaike Information Criterion (AIC) because model selection via AIC led to inconsistent results during sensitivity analyses (see below).

For some habitat covariates, observations were missing due to equipment malfunction, equipment loss, or technician error (***SI Appendix,*** **Table S5**). Removing observations with only complete information limited the amount of data available for modeling. Therefore, two approaches were used to account for missing values of habitat covariates to evaluate the sensitivity of model selection to alternative approaches: (*i*) a likelihood-based approach (***SI***

Appendix,* Table S6**) and (*ii*) an imputation approach (SI Appendix,* Table S7**; 39). For the likelihood-based approach, missing continuous covariates were filled in with a value drawn from a normal distribution with a mean of 0 and variance of 1. An analogous approach was used to account for missing categorical covariates. Let $\mathbf{x}_i = (\mathbf{x}_{i,obs}, \mathbf{x}_{i,mis})$, where $\mathbf{x}_{i,obs}$ and $\mathbf{x}_{i,mis}$ represent the observed and missing categorical components of $\mathbf{x}_i$ for each observation, respectively. The probability function (Eqs. 1–7) was then marginalized across all possible combinations of $\mathbf{x}_{i,mis}$:

$$f(c_i|\mathbf{x}_{\boldsymbol{i}}, \theta_i) = \sum_{\mathbf{x}_{i,mis}(j)} f\left(c_i|\mathbf{x}_{i,obs}, \mathbf{x}_{i,mis}(j), \theta_i\right) p\left(\mathbf{x}_{i,mis}(j)|\mathbf{x}_{i,obs}, \boldsymbol{\alpha}\right)$$

where $\theta = (ß_{0,p}, ß_{0,c}, ß_p, ß_c, \sigma_{s,p}^2, \sigma_{s,c}^2, \sigma_c^2)$, j indexes the distinct combination of $\mathbf{x}_{i,mis}$, and α_j is a vector of parameters (i.e., Bernoulli probabilities) for the jth conditional distribution estimated from the observed data. For the imputation approach, the mean and mode were imputed for missing continuous and categorical variables, respectively. Given the potential influence of outliers on model selection, both approaches were also conducted on a reduced dataset where an observation (from Mbakhana in May 2016) with 901 snails was removed. However, removal led to qualitatively similar results, suggesting that model selection and fit were robust to outliers. All models were fit using Template Model Builder in the R Statistical Environment (40).

Observed versus predicted values indicate that the best-fitting model adequately captured variation in snail density (***SI Appendix,* Figure S6a–b**).

***SI Appendix,* Text S8. Detailed methods: calculating site-level characteristics**

Images of sites from each field mission were analyzed in Google Earth Pro (Google Inc., version 7.3) and ArcGIS (ESRI 2011. ArcGIS Desktop: Release 10. Redlands, CA: Environmental Systems Research Institute). We defined the boundaries of the total site area as we had defined the sampling boundary (see above), except where the sampling boundary's offshore edge matched the 100-cm depth isocline, the total site area boundary's offshore edge was either at the 150-cm depth isocline or 50 m from shore, whichever was closer. This edge was chosen because it encompasses most human activity within the site (i.e., swimmers infrequently pass this boundary), and therefore encompasses the relevant area for human contact with schistosome cercariae. The sampling boundary was a subset of the total site area boundary because it was unsafe for technicians to work in water >100 cm deep.

Using aerial imagery, technicians who were extensively trained and actively involved in the identification and monitoring of aquatic vegetation at the sampling sites visually classified the microhabitats and calculated the total area, area of mud, area of emergent vegetation, and area of non-emergent vegetation within the total site area boundary for each field mission (using Google Earth Pro and ArcGIS; **Figure 1D, main text**). Because human infection data were collected at the level of the village, and because we wished to understand how habitat characteristics influenced patterns of human infection, we aggregated site-level data to the village level (for those villages that contained >1 site). We first calculated all characteristics at the site level for each field mission. We then aggregated these site-level estimates to the village level for each field mission by taking the weighted mean, where weight was equal to the area of the site. Finally, because we assume that risk factors integrate over time, we summed across field missions.

***SI Appendix,* Table S1.** Hypothesized predictors of human schistosomiasis burden included in analyses of site-level predictors of human schistosomiasis burden.

**Predictor**	**Calculation**	**Hypothesis**
(1) Snail density (weighted average across all sites in village; if multiple sites are present in a village, then density is weighted by total size of individual sites)	∑ [[snails/m^2 per habitat type × (% habitat type)] × Area of site (10), divided by total area of all sites associated with that village]	The greater the density of snails, the likelier that group of snails is to contain some infected individuals. If a village contained multiple sites, snail density was calculated as a weighted mean across sites (weighted by site size), based on the assumption that people visit larger sites more frequently than smaller sites, which are more commonly used by fishermen.
(2) Snail abundance (snail density as weighted average across sites by site size × total area of all sites)	Snail density (1) × Area of site (10)	The higher the absolute abundance of susceptible snails, the higher the likelihood of transmission from humans to snails.
(3) *S. haematobium* prevalence in snails	∑ [[infected snails/m^2 per habitat type] ÷ [all snails/m^2 per habitat type]]	Human infection burden will increase with increasing availability of *S. haematobium* cercariae in the environment, which will increase with increasing proportion of snails infected with *S. haematobium*. *S. haematobium* prevalence in snails was calculated as a weighted mean of prevalence within discrete habitat types, weighted by the amount of habitat type sampled.
(4) Infected snail density	Snail density (1) × *S. haematobium* prevalence in snails (3)	Human infection burden will increase with increasing availability of *S. haematobium* cercariae in the environment, which will increase with increasing density of snails infected with *S. haematobium*. Infected snail density should be a better predictor of the availability of *S. haematobium* cercariae than is *S. haematobium* prevalence (3), because it adjusts risk for the number of snails per m^2.
(5) Infected snail abundance	Snail abundance (2) × *S. haematobium* prevalence in snails	Human infection burden will increase with increasing availability of *S. haematobium* cercariae in the

	(3)	environment, which will increase with increasing abundance of snails infected with *S. haematobium*. Infected snail abundance should be a better predictor of the availability of *S. haematobium* cercariae than is infected snail density (4), because it adjusts risk for the number of snails at the entire site.
(6) Area (%) of suitable snail habitat	% of site covered by non-emergent vegetation, as measured in ArcGIS using photos taken from unmanned aerial vehicle (drone) at the time of snail sampling	Human infection burden will increase with increasing proportion of suitable snail habitat, because increasing suitable snail habitat increases the likelihood of snail presence and abundance, which increases the likelihood that a group of snails will contain some infected individuals.
(7) Area (%) of water contact sites dominated by mud	% of site covered by mud, as measured in ArcGIS using photos taken from unmanned aerial vehicle (drone) at the time of snail sampling	Human infection burden will increase with proportion of mud, because such open habitat is available for colonization by the aquatic vegetation species used by snails. Over time, mud habitat may convert to suitable snail habitat; this increases the availability of suitable snail habitat when integrating over time, which increases the likelihood of snail presence and abundance, which increases the likelihood that a group of snails will contain some infected individuals.
(8) Area (m^2) of suitable snail habitat	Area (%) of suitable snail habitat (6) × Area of site (m^2) (10)	Human infection burden will increase with increasing area of suitable snail habitat, because increasing suitable snail habitat increases the likelihood of snail presence and abundance, which increases the likelihood that a group of snails will contain some infected individuals. Area of suitable snail habitat should be a better predictor of the availability of *S. haematobium* cercariae than is proportion of suitable snail habitat (6), because it adjusts risk for the area of the entire site.
(9) Area (m^2) of village water contact sites dominated by mud	Area (%) of mud (7) × Area of site (m^2) (10)	Human infection burden will increase with area of mud, because such open habitat is available for colonization by

		the aquatic vegetation species used by snails. Over time, mud habitat may convert to suitable snail habitat; this increases the availability of suitable snail habitat when integrating over time, which increases the likelihood of snail presence and abundance, which increases the likelihood that a group of snails will contain some infected individuals. Area of site covered by mud should be a better predictor of the availability of *S. haematobium* cercariae than is proportion of site covered in mud (8), because it adjusts risk for the area of the entire site.
(10) Area of site (m^2)	area of site, as measured as measured in ArcGIS using photos taken from unmanned aerial vehicle (drone) at the time of snail sampling	Human infection burden will increase with area of site, because larger sites contain more area available for colonization by the aquatic vegetation species used by snails. This increases the availability of suitable snail habitat when integrating over time, which increases the likelihood of snail presence and abundance, which increases the likelihood that a group of snails will contain some infected individuals.
Demographic predictors	sex, age, & village population size	

***SI Appendix,* Table S2.** Comparison of BIC and MSE for all seven models aiming to identify snail and snail–habitat predictors of human urogenital schistosomiasis burden. These results are from models that used only snail-related variables (i.e., snail and snail–habitat variables, no habitat variables) to predict human infection burden, and are described in main text under the headings, *Identifying snail-related predictors of human urogenital schistosomiasis burden*. (a) Logistic model of likelihood of human infection and (b) negative binomial model of human egg count. All models include demographic variables (age, sex, village population, and location on lake or river) and random effects (village and individual ID). ∆BIC = delta BIC = difference in BIC from most well-supported model (i.e., the model with the lowest BIC = 1726.26 for [a] logistic and 17.27 for [b] negative binomial) and ∆MSE = delta mean squared error = difference in MSE from most well-supported model (i.e., the model with the lowest MSE = 0.17 for [a] logistic and 566.84 for [b] negative binomial). A permutation test for Moran's I statistic was calculated to assess spatial-autocorrelation among villages. We used 999 permutations of the village-level residuals from each model, given a spatial weighting scheme based on inverse distance among villages. Below, we show the value of the Moran's I statistic, its rank among the 999 permutations, and the corresponding pseudo p-value of the test. Note that all snail variables are habitat-corrected (see ***SI Appendix,* Table S1**).

(a)

**model rank (and number in *SI Appendix* Figure 4a)**	**model specification**	**∆BIC**	**BIC weight**	**Moran's *I* statistic**	**Moran's *I* observed rank**	**Moran's *I* p-value**	**∆MSE**
1	null model (including only demographic predictors and random effects)	0.00	0.63	0.10481	835	0.165	0.0014
2	snail abundance	2.19	0.21	0.13146	835	0.165	0.0000
3	infected snail abundance	3.74	0.10	0.10475	817	0.183	0.0013
4	snail density	6.64	0.02	0.1283	849	0.151	0.0014
5	snail prevalence	6.95	0.02	0.10465	855	0.145	0.0019
6	infected snail density	7.37	0.02	0.12365	849	0.151	0.0021
7	snail density + snail prevalence	12.67	0.00	0.13945	842	0.158	0.0019

(b)

model rank (and number in *SI Appendix* Figure 4b)	model specification	∆BIC	BIC weight	Moran's *I* statistic	Moran's *I* observed rank	Moran's *I* p-value	MSE
1	snail abundance	0.00	0.63	0.02990	736	0.264	137.85
2	infected snail abundance	1.09	0.37	-0.03589	650	0.350	0.00
3	null model (including only demographic predictors and random effects)	12.40	0.00	-0.05628	583	0.417	332.10
4	snail density + snail prevalence	14.02	0.00	0.01488	699	0.301	232.86
5	infected snail density	15.52	0.00	-0.06738	601	0.399	249.89
6	snail density	15.53	0.00	-0.00961	666	0.334	320.67
7	snail prevalence	15.75	0.00	-0.04733	600	0.400	282.62

***SI Appendix,* Table S3.** Results of the logistic GLMM using exclusively snail and snail–habitat predictors. All three models within 10 ΔBIC of the top model are shown here. Models are numbered by their BIC rank and are described in ***SI Appendix,* Table S2a**. An odds ratio > 1 indicates a positive influence of the covariate on human infection burden, and an odds ratio < 1 indicates a negative influence of the covariate on human infection burden. Ranges in parentheses indicate 95% confidence intervals. All models included a random effect of individual identity nested within village; σ^2 = within-group variance of random effects and τ_{00} = between-group variance of random effects. Demographic predictors are listed at the top of the table, followed by snail–habitat and snail predictors. These results are from models that used only snail and snail–habitat variables (i.e., no habitat variables) to predict human infection burden, and are described in main text under the heading, *Identifying snail-related predictors of human urogenital schistosomiasis burden*. Note that all snail variables are habitat-corrected (see ***SI Appendix,* Table S1**).

	Model 1	***Model 2***	***Model 3***	***Model 4***	***Model 5***	***Model 6***
Predictors	***Odds ratio (CI)***	***Odds ratio (CI)***	***Odds ratio (CI)***	***Odds ratio (CI)***	***Odds ratio (CI)***	***Odds ratio (CI)***
(Intercept)	0.49 (0.20 – 1.22)	0.42 (0.19 – 0.91)	0.49 (0.21 – 1.17)	0.40 (0.17 – 0.97)	0.49 (0.20 – 1.22)	0.45 (0.18 – 1.10)
Location: lake (vs river)	12.95 (4.37 – 38.38)	17.52 (6.91 – 44.45)	13.16 (4.69 – 36.93)	19.42 (6.57 – 57.37)	12.92 (4.35 – 38.37)	12.91 (4.40 – 37.83)
Sex: male (vs female)	1.45 (1.11 – 1.90)	1.45 (1.11 – 1.90)	1.45 (1.11 – 1.90)	1.46 (1.11 – 1.90)	1.45 (1.11 – 1.90)	1.45 (1.11 – 1.90)
Village population	0.73 (0.44 – 1.22)	0.82 (0.55 – 1.23)	0.72 (0.44 – 1.18)	0.81 (0.51 – 1.28)	0.73 (0.44 – 1.22)	0.90 (0.55 – 1.48)
School grade	0.93 (0.81 – 1.07)	0.93 (0.81 – 1.07)	0.93 (0.81 – 1.07)	0.93 (0.81 – 1.08)	0.93 (0.81 – 1.07)	0.94 (0.81 – 1.08)
Snail abundance		1.57 (1.24 – 2.00)				
Infected snail abundance			1.11 (0.88 – 1.39)			
Snail density				1.59 (1.15 – 2.20)		
Snail prevalence					0.99 (0.86 – 1.16)	
Infected snail density						0.75 (0.59 – 0.95)
**Random effects**						
σ^2	3.29	3.29	3.29	3.29	3.29	3.29
τ_{00}	0.59 $_{ID:village}$	0.62 $_{ID:village}$	0.60 $_{ID:village}$	0.60 $_{ID:village}$	0.59 $_{ID:village}$	0.59 $_{ID:village}$
	0.67 $_{village}$	0.45 $_{village}$	0.59 $_{village}$	0.61 $_{village}$	0.67 $_{village}$	0.66 $_{village}$

***SI Appendix,* Table S4.** Results of the negative binomial GLMM using exclusively snail and snail–habitat predictors. Both models within 10 ΔBIC of the top model are shown here. Models are numbered by their BIC rank and are described in ***SI Appendix,* Table S2b**. An incidence rate ratio > 1 indicates a positive influence of the covariate on human infection burden, and an incidence rate ratio < 1 indicates a negative influence of the covariate on human infection burden. Ranges in parentheses indicate 95% confidence intervals. All models included a random effect of individual identity nested within village; σ^2 = within-group variance of random effects and τ_{00} = between-group variance of random effects. Demographic predictors are listed at the top of the table, followed by snail–habitat predictors. These results are from models that used only snail and snail–habitat variables (i.e., no habitat variables) to predict human infection burden, and are described in main text under the heading, *Identifying snail-related predictors of human urogenital schistosomiasis burden*. Note that all snail variables are habitat-corrected (see ***SI Appendix,* Table S1**).

	Model 1	***Model 2***
Predictors	***Incidence rate ratio (CI)***	***Incidence rate ratio (CI)***
(Intercept)	1.31 (0.73 – 2.34)	1.39 (0.73 – 2.64)
Location: lake (vs river)	35.02 (18.81 – 65.20)	28.75 (14.17 – 58.35)
Sex: male (vs female)	1.62 (1.24 – 2.12)	1.61 (1.23 – 2.10)
Village population	0.97 (0.72 – 1.29)	1.02 (0.73 – 1.43)
School grade	0.68 (0.60 – 0.78)	0.69 (0.61 – 0.79)
Snail abundance	1.79 (1.41 – 2.28)	
Infected snail abundance		1.27 (1.14 – 1.41)
**Random effects**		
σ^2	1.21	1.21
τ_{00}	2.09 $_{ID:Village}$	2.10 $_{ID:Village}$
	0.20 $_{Village}$	0.29 $_{Village}$

***SI Appendix,* Table S5.** Summary of habitat covariates considered for model selection of delta-Poisson-lognormal regression models of the count of snail *Bulinus truncatus/globosus*. The mean is noted for continuous variables and the mode for categorical.

Category	Covariate	Units/type	Mean/mode	SD	% missing
Water quality variables	pH		7.29	0.37	10.09%
	Secchi depth	cm	43.37	12.21	0.78%
	water depth	cm	37.09	17.65	0.10%
	NO_2	mg l^{-1}	0.06	0.33	1.56%
	NO_3	mg l^{-1}	2.32	1.51	15.61%
	PO_4	mg l^{-1}	0.39	0.47	3.12%
	river- or lake-dominated	Categorical; river = 0; lake = 1	1	NA	0.00%
Non-emergent vegetation variables	cumulative mass of floating vegetation	g	199.76	455.07	0.36%
	Cyperus	Categorical; presence/absence	0	NA	0.10%
	Poa	Categorical; presence/absence	0	NA	0.10%
	Ludwigia	Categorical; presence/absence	0	NA	0.10%
	Nymphaea	Categorical; presence/absence	0	NA	0.10%
	Salvinia	Categorical; presence/absence	0	NA	0.10%
	Ceratophyllum	Categorical; presence/absence	0	NA	0.10%
	Potamogeton	Categorical; presence/absence	0	NA	0.10%
	Pistia	Categorical; presence/absence	0	NA	0.10%
	Azolla	Categorical; presence/absence	0	NA	23.99%
	Ipomea	Categorical; presence/absence	0	NA	23.99%
	Lemna	Categorical; presence/absence	0	NA	65.19%
Emergent vegetation variables	cumulative number of emergent stems	number	1.29	4.85	0.26%
	Typha	Categorical; presence/absence	0	NA	0.16%
	Phragmitesmites	Categorical; presence/absence	0	NA	0.10%
Interaction terms	Cumulative mass of floating vs cumulative number of emergent stems		257.20	1,857.74	0.52%

***SI Appendix,* Table S6.** Forward and backward model selection of delta-Poisson-lognormal regression models relating the count of snail *Bulinus truncatus/globosus* to habitat covariates, **where a likelihood based approach was used to substitute missing covariates**. Negative log-likelihood (NLL), number of parameters (K), and Bayesian information criterion (BIC) of each model are included. Final model is included in the last row.

Covariates included	NLL	K	BIC	∆BIC	BIC weight
**Forward selection**					
Intercept only	9,123	19	18,389	387	0.00
Secchi depth	9,119	21	18,396	394	0.00
pH	9,122	21	18,403	401	0.00
Nitrate	9,119	21	18,396	394	0.00
Nitrite	9,122	21	18,403	401	0.00
Phosphate	9,121	21	18,401	399	0.00
Water depth	9,114	21	18,387	385	0.00
Water depth, emergent stems	9,109	23	18,392	390	0.00
Water depth, non-emergent vegetation	8,984	23	18,142	140	0.00
Water depth, non-emergent vegetation, non-emergent vs emergent	8,984	25	18,157	155	0.00
Water depth, non-emergent vegetation, *Typha*	8,979	25	18,147	145	0.00
Water depth, non-emergent vegetation, *Phragmites*	8,984	25	18,157	155	0.00
Water depth, non-emergent vegetation, *Cyperus*	8,984	25	18,157	155	0.00
Water depth, non-emergent vegetation, *Poa*	8,984	25	18,157	155	0.00
Water depth, non-emergent vegetation, *Ludwigia*	8,971	25	18,132	130	0.00
Water depth, non-emergent vegetation, *Ludwigia*, *Nymphaea*	8,970	27	18,144	142	0.00
Water depth, non-emergent vegetation, *Ludwigia*, *Salvinia*	8,968	27	18,140	138	0.00
Water depth, non-emergent vegetation, *Ludwigia*, *Ceratophyllum*	8,920	27	18,045	43	0.00
Water depth, non-emergent vegetation, *Ludwigia*, *Ceratophyllum*, *Potamogeton*	8,902	29	18,024	22	0.00
Water depth, non-emergent vegetation, *Ludwigia*, *Ceratophyllum*, *Potamogeton*, *Pistia*	8,899	31	18,031	29	0.00
Water depth, non-emergent vegetation, *Ludwigia*, *Ceratophyllum*, *Potamogeton*, *Azolla*	8,897	31	18,029	27	0.00
Water depth, non-emergent vegetation, *Ludwigia*, *Ceratophyllum*, *Potamogeton*, *Ipomea*	8,902	31	18,039	37	0.00
Water depth, non-emergent vegetation, *Ludwigia*, *Ceratophyllum*, *Potamogeton*, *Lemna*	8,902	31	18,039	37	0.00
Water depth, non-emergent vegetation, *Ludwigia*, *Ceratophyllum*, *Potamogeton*, lake/river	8,890	31	18,015	13	0.00
**Backward selection**					
Non-emergent vegetation, *Ludwigia*, *Ceratophyllum*, *Potamogeton*, lake/river	8,892	29	18,002	0	0.87
Ludwigia, *Ceratophyllum*, *Potamogeton*, lake/river	8,975	27	18,154	152	0.00
Non-emergent vegetation, *Ceratophyllum*, *Potamogeton*, lake/river	8,901	27	18,006	4	0.12
Non-emergent vegetation, *Ludwigia*, *Potamogeton*, lake/river	8,940	27	18,084	82	0.00
Non-emergent vegetation, *Ludwigia*, *Ceratophyllum*, lake/river	8,915	27	18,033	31	0.00
Non-emergent vegetation, *Ludwigia*, *Ceratophyllum*, *Potamogeton*	8,904	27	18,012	10	0.01
Non-emergent vegetation, *Ludwigia*, *Ceratophyllum*, *Potamogeton*, lake/river	8,892	29	18,002	0	0.87

***SI Appendix,* Table S7.** Forward and backward model selection of delta-Poisson-lognormal regression models relating the count of snail *Bulinus truncatus/globosus* to habitat covariates, **where the mean or mode were imputed for missing covariates**. Negative log-likelihood (NLL), number of parameters (K), and Bayesian information criterion (BIC) of each model are included. Final model is included in the last row.

**Covariates included**	**NLL**	**K**	**BIC**	**ΔBIC**	**BIC weight**
**Forward selection**					
Intercept only	9,153	19	18,450	387	0.00
Secchi depth	9,150	21	18,458	395	0.00
pH	9,153	21	18,465	402	0.00
Nitrate	9,149	21	18,458	395	0.00
Nitrite	9,153	21	18,465	402	0.00
Phosphate	9,152	21	18,463	400	0.00
Water depth	9,145	21	18,449	386	0.00
Water depth, emergent stems	9,140	23	18,454	391	0.00
Water depth, non-emergent vegetation	9,015	23	18,204	141	0.00
Water depth, non-emergent vegetation, non-emergent vs emergent	9,015	25	18,219	156	0.00
Water depth, non-emergent vegetation, *Typha*	9,010	25	18,209	146	0.00
Water depth, non-emergent vegetation, *Phragmites*	9,015	25	18,219	156	0.00
Water depth, non-emergent vegetation, *Cyperus*	9,015	25	18,219	156	0.00
Water depth, non-emergent vegetation, *Poa*	9,014	25	18,218	155	0.00
Water depth, non-emergent vegetation, *Ludwigia*	9,002	25	18,193	130	0.00
Water depth, non-emergent vegetation, *Ludwigia*, *Nymphaea*	9,000	27	18,205	142	0.00
Water depth, non-emergent vegetation, *Ludwigia*, *Salvinia*	8,998	27	18,201	138	0.00

Covariates included	NLL	K	BIC	∆BIC	BIC weight
**Forward selection (cont'd)**					
Water depth, non-emergent vegetation, *Ludwigia*, *Ceratophyllum*	8,951	27	18,105	42	0.00
Water depth, non-emergent vegetation, *Ludwigia*, *Ceratophyllum*, *Potamogeton*	8,933	29	18,085	22	0.00
Water depth, non-emergent vegetation, *Ludwigia*, *Ceratophyllum*, *Potamogeton*, *Pistia*	8,929	31	18,092	29	0.00
Water depth, non-emergent vegetation, *Ludwigia*, *Ceratophyllum*, *Potamogeton*, *Azolla*	8,927	31	18,087	24	0.00
Water depth, non-emergent vegetation, *Ludwigia*, *Ceratophyllum*, *Potamogeton*, *Ipomea*	8,933	31	18,099	36	0.00
Water depth, non-emergent vegetation, *Ludwigia*, *Ceratophyllum*, *Potamogeton*, *Lemna*	8,933	31	18,100	37	0.00
Water depth, non-emergent vegetation, *Ludwigia*, *Ceratophyllum*, *Potamogeton*, lake/river	8,921	31	18,076	13	0.00
**Backward selection**					
Non-emergent vegetation, *Ludwigia*, *Ceratophyllum*, *Potamogeton*, lake/river	8,922	29	18,063	0	0.87
Ludwigia, *Ceratophyllum*, *Potamogeton*, lake/river	9,006	27	18,216	153	0.00
Non-emergent vegetation, *Ceratophyllum*, *Potamogeton*, lake/river	8,932	27	18,067	4	0.12
Non-emergent vegetation, *Ludwigia*, *Potamogeton*, lake/river	8,971	27	18,146	83	0.00
Non-emergent vegetation, *Ludwigia*, *Ceratophyllum*, lake/river	8,945	27	18,094	31	0.00
Non-emergent vegetation, *Ludwigia*, *Ceratophyllum*, *Potamogeton*	8,934	27	18,073	10	0.01
Non-emergent vegetation, *Ludwigia*, *Ceratophyllum*, *Potamogeton*, lake/river	8,922	29	18,063	0	0.87

***SI Appendix,* Table S8.** Parameter estimates from best-fit delta-Poisson-lognormal regression model relating the count of snail *Bulinus truncatus/globosus* to habitat covariates, where a likelihood-based approach was used to substitute missing covariates. The partial response is the expected count (i.e., the product of encounter probability and expected non-zero count) predicted for 1 unit increase of each covariate while holding all other covariates at zero.

	**Probability of zero counts (*p*)**		**Non-zero counts (*c*)**		
**Covariate**	**Estimate**	**SE**	**Estimate**	**SE**	**Partial response**
Intercept	2.123	0.305	0.958	0.228	2.328
Regression coefficient of non-emergent vegetation mass	-0.653	0.074	0.400	0.045	3.660
Regression coefficient of *Ludwigia* presence/absence	-0.635	0.170	0.344	0.146	3.457
Regression coefficient of *Ceratophyllum* presence/absence	-1.618	0.171	0.359	0.173	3.646
Regression coefficient of *Potamogeton* presence/absence	-1.511	0.230	0.295	0.199	3.411
Regression coefficient of river- or lake-dominated ecosystem (river = 0; lake =1)	1.442	0.355	-0.824	0.220	0.759
Variance of random site effect	0.728	0.176	0.314	0.121	
Variance of overdispersion parameterization			1.029	0.053	

***SI Appendix*, Table S9.** Estimated site-specific random effects and associated partial response (holding all habitat covariates at zero) from the best-fit delta-Poisson-lognormal regression model relating the count of snail *Bulinus truncatus/globosus* to habitat covariates, where a likelihood-based approach was used to substitute missing covariates.

Site	$\varepsilon_{s,p}$	$\varepsilon s, c$	Partial response
Global average	0.000	0.000	2.328
Diokhor 1	-0.877	-0.430	1.317
Diokhor 2	-0.947	-0.461	1.256
Diokoul 1	0.218	-0.889	0.977
Diokoul 2	-0.093	0.407	3.463
Foss	0.653	-0.266	1.881
Gankette	0.044	-0.345	1.656
Guidik 1	-0.091	0.581	4.119
Guidik 2	0.629	-0.846	1.051
Guidik 3	0.609	0.375	3.562
Guidik 4	0.127	-0.468	1.477
Lampsar 1	0.282	-0.121	2.119
Lampsar 2	0.216	-0.063	2.232
Maka Diama 1	0.727	1.236	8.478
Maka Diama 2	0.744	0.344	3.478
Malla	-0.286	-0.210	1.822
Malla Tack 1	-0.484	-1.176	0.674
Malla Tack 2	0.327	-0.203	1.958
Malla Tack 3	0.298	0.054	2.528
Mbakhana	0.000	0.000	2.328
Mbane 1	0.073	0.055	2.478
Mbane 3	0.079	-0.094	2.136
Mbarigot 1	-0.281	-0.084	2.068
Mbarigot 2	0.163	-0.145	2.046
Mbarigot 3	0.000	0.244	2.970
Merina Gewel 1	0.088	-0.158	2.006
Merina Gewel 2	0.369	-0.202	1.967
Merina Gewel 3	0.000	-0.190	1.926
Merina Gewel 4	0.437	0.299	3.262
Ndiawdoune	0.000	0.000	2.328
Ndiol Maure	0.000	-0.060	2.192
Syer 1	-0.004	-0.143	2.017
Syer 2	0.049	0.276	3.082

***SI Appendix,* Table S10.** Comparison of BIC for all 14 models for (a) logistic model of likelihood of human infection and (b) negative binomial model of human egg count. These results are from models that snail, snail–habitat, and habitat variables to predict human infection burden, and are described in main text under the headings, *Identifying snail- and habitat-related predictors of human urogenital schistosomiasis burden*. Models that include exclusively snail-related predictors are highlighted. All models include demographic variables (age, sex, village population, and location on lake or river) and random effects (village and individual ID). ∆BIC = delta BIC = difference in BIC from most well-supported model (i.e., the model with the lowest BIC = 1720.52 for [a] logistic and 11609.95 for [b] negative binomial) and ∆MSE = delta mean squared error = difference in MSE from most well-supported model (i.e., the model with the lowest MSE = 0.17 for [a] logistic and 7505.22 for [b] negative binomial). A permutation test for Moran's I statistic was calculated to assess spatial-autocorrelation among villages. We used 999 permutations of the village-level residuals from each model, given a spatial weighting scheme based on inverse distance among villages. Below, we show the value of the Moran's I statistic, its rank among the 999 permutations, and the corresponding pseudo p-value of the test. Note that all snail variables are habitat-corrected (see ***SI Appendix,* Table S1**).

(a)

model rank (and number in Figure 4a)	model specification	∆BIC	Moran's *I* statistic	Moran's *I* observed rank	Moran's *I* p-value	∆MSE
1	total area of non-emergent vegetation + total area of mud	0.00	0.06100	792	0.208	0.0000
2	null model (including only demographic predictors and random effects)	5.74	0.10481	835	0.165	0.0059
3	area of site + percent area of non-emergent vegetation + percent area of mud	7.04	0.09191	841	0.159	0.0010
4	snail abundance	7.93	0.13146	835	0.165	0.0045
5	total mass of non-emergent vegetation	8.58	0.12443	834	0.166	0.0041
6	infected snail abundance	9.48	0.10475	817	0.183	0.0058
7	site area + snail density	10.32	0.11889	840	0.160	0.0023
8	snail density	12.38	0.12830	849	0.151	0.0059
9	snail prevalence	12.69	0.10465	855	0.145	0.0064
10	infected snail density	13.11	0.12365	849	0.151	0.0066
11	area of site + percent area of non-emergent vegetation + percent area of mud + average mass of non-emergent vegetation sampled	13.69	0.10793	826	0.174	0.0013
12	site area + snail density + snail prevalence	16.69	0.13339	850	0.150	0.0029
13	snail density + snail prevalence	18.41	0.13945	842	0.158	0.0064
14	full orthogonal model: site size + average mass of non-emergent vegetation + snail density + snail infection prevalence + percent area of non-emergent vegetation + percent area of mud	28.40	0.11219	824	0.176	0.0015

(b)

model rank (and number in Figure 4b)	model specification	ΔBIC	Moran's *I* statistic	Moran's *I* observed rank	Moran's *I* p-value	ΔMSE
1	total area of non-emergent vegetation + total area of mud	0.00	-0.16773	396	0.604	129.47
2	area of site + percent area of non-emergent vegetation + percent area of mud	2.08	-0.14711	443	0.557	66.28
3	area of site + percent area of non-emergent vegetation + percent area of mud + average mass of non-emergent vegetation sampled	2.33	-0.04108	598	0.402	59.78
4	total mass of non-emergent vegetation	8.94	-0.04319	641	0.359	665.59
5	full orthogonal model: site size + average mass of non-emergent vegetation + snail density + snail prevalence + percent area of non-emergent vegetation + percent of mud	9.20	-0.06965	587	0.413	0.00
6	snail abundance	17.27	0.02990	736	0.264	704.68
7	infected snail abundance	18.36	-0.03589	650	0.35	566.84
8	site area + snail density + snail prevalence	24.43	-0.00725	684	0.316	236.17
9	site area + snail density	25.72	-0.04735	605	0.395	366.37
10	null model (including only demographic predictors and random effects)	29.67	-0.05628	583	0.417	898.94
11	snail density + snail prevalence	31.29	0.01488	699	0.301	799.70
12	infected snail density	32.79	-0.06738	601	0.399	816.73
13	snail density	32.80	-0.00961	666	0.334	887.51
14	snail prevalence	33.02	-0.04733	600	0.4	849.46

***SI Appendix,* Table S11.** Results of the logistic GLMM using snail, snail–habitat, and habitat variables. All six models within 10 ΔBIC of the top model are shown here. Models are numbered by their BIC rank and are described in ***SI Appendix,* Table S12a**. An odds ratio > 1 indicates a positive influence of the covariate on human infection burden, and an odds ratio < 1 indicates a negative influence of the covariate on human infection burden. Ranges in parentheses indicate 95% confidence intervals. All models included a random effect of individual identity nested within village; σ^2 = within-group variance of random effects and τ_{00} = between-group variance of random effects. Demographic predictors are listed at the top of the table, followed by habitat variables, snail–habitat variables, and finally snail variables. These results are from models that used both snail, snail–habitat, and habitat variables to predict human infection burden, and are described in main text under the heading, *Identifying snail- and habitat-related predictors of human urogenital schistosomiasis burden*. Note that all snail variables are habitat-corrected (see ***SI Appendix,* Table S1**).

	Model 1	***Model 2***	***Model 3***	***Model 4***	***Model 5***	***Model 6***
Predictors	***Odds ratio (CI)***	***Odds ratio (CI)***	***Odds ratio (CI)***	***Odds ratio (CI)***	***Odds ratio (CI)***	***Odds ratio (CI)***
(Intercept)	0.70<br>(0.38 – 1.27)	0.49<br>(0.20 – 1.22)	0.66<br>(0.37 – 1.18)	0.42<br>(0.19 – 0.91)	0.50<br>(0.24 – 1.04)	0.49<br>(0.21 – 1.17)
Location: lake (vs river)	7.31<br>(3.55 – 15.05)	12.95<br>(4.37 – 38.38)	8.54<br>(4.24 – 17.21)	17.52<br>(6.91 – 44.45)	10.63<br>(4.46 – 25.33)	13.16<br>(4.69 – 36.93)
Sex: male (vs female)	1.48<br>(1.11 – 1.95)	1.45<br>(1.11 – 1.90)	1.49<br>(1.12 – 1.98)	1.45<br>(1.11 – 1.90)	1.46<br>(1.12 – 1.91)	1.45<br>(1.11 – 1.90)
Village population	0.77<br>(0.57 – 1.06)	0.73<br>(0.44 – 1.22)	0.65<br>(0.46 – 0.92)	0.82<br>(0.55 – 1.23)	0.71<br>(0.48 – 1.05)	0.72<br>(0.44 – 1.18)
School grade	0.92<br>(0.79 – 1.07)	0.93<br>(0.81 – 1.07)	0.92<br>(0.79 – 1.07)	0.93<br>(0.81 – 1.07)	0.94<br>(0.82 – 1.08)	0.93<br>(0.81 – 1.07)
Site area			1.68<br>(1.22 – 2.32)			
Area of non-emergent vegetation	2.46<br>(1.62 – 3.74)					
Area of mud	0.99<br>(0.71 – 1.40)					
Percent cover of non-emergent vegetation			1.79<br>(1.33 – 2.40)			
Percent cover of mud			1.04<br>(0.88 – 1.24)			

	Model 1	Model 2	Model 3	Model 4	Model 5	Model 6
Predictors	***Odds ratio (CI)***	***Odds ratio (CI)***	***Odds ratio (CI)***	***Odds ratio (CI)***	***Odds ratio (CI)***	***Odds ratio (CI)***
Mass of non-emergent vegetation					1.47 (1.16 – 1.86)	
Snail abundance				1.57 (1.24 – 2.00)		
Infected snail abundance						1.11 (0.88 – 1.39)
**Random effects**						
σ^2	3.29	3.29	3.29	3.29	3.29	3.29
τ_{00}	0.69 $_{ID:village}$	0.59 $_{ID:village}$	0.72 $_{ID:village}$	0.62 $_{ID:village}$	0.62 $_{ID:village}$	0.60 $_{ID:village}$
	0.18 $_{village}$	0.67 $_{village}$	0.17 $_{village}$	0.45 $_{village}$	0.39 $_{village}$	0.59 $_{village}$

***SI Appendix,* Table S12.** Results of the negative binomial GLMM using snail, snail–habitat, and habitat predictors. All five models within 10 ∆BIC of the top model are shown here. Models are numbered by their BIC rank and are described in ***SI Appendix,* Table S12b**. An incidence rate ratio > 1 indicates a positive influence of the covariate on human infection burden, and an incidence rate ratio < 1 indicates a negative influence of the covariate on human infection burden. Ranges in parentheses indicate 95% confidence intervals. All models included a random effect of individual identity nested within village; σ^2 = within-group variance of random effects and τ_{00} = between-group variance of random effects. Demographic predictors are listed at the top of the table, followed by habitat variables, snail–habitat variables, and finally snail variables. These results are from models that used snail, snail–habitat, habitat variables to predict human infection burden, and are described in main text under the heading, *Identifying snail- and habitat-related predictors of human urogenital schistosomiasis burden*. Note that all snail variables are habitat-corrected (see ***SI Appendix,* Table S1**).

	Model 1	***Model 2***	***Model 3***	***Model 4***	***Model 5***
Predictors	***Incidence rate ratio (CI)***	***Incidence rate ratio (CI)***	***Incidence rate ratio (CI)***	***Incidence rate ratio (CI)***	***Incidence rate ratio (CI)***
(Intercept)	2.09 (1.38 – 3.18)	2.02 (1.33 – 3.08)	2.03 (1.33 – 3.08)	1.78 (0.98 – 3.22)	2.01 (1.29 – 3.14)
Location: lake (vs river)	15.53 (10.86 – 22.20)	17.15 (11.98 – 24.55)	15.34 (10.62 – 22.16)	15.43 (8.18 – 29.12)	14.95 (8.87 – 25.19)
Sex: male (vs female)	1.64 (1.25 – 2.14)	1.68 (1.28 – 2.21)	1.70 (1.29 – 2.22)	1.66 (1.27 – 2.17)	1.70 (1.30 – 2.23)
Village population	0.96 (0.83 – 1.11)	0.78 (0.66 – 0.93)	0.69 (0.57 – 0.84)	0.74 (0.56 – 0.99)	0.70 (0.58 – 0.85)
School grade	0.66 (0.58 – 0.76)	0.66 (0.58 – 0.75)	0.66 (0.58 – 0.76)	0.70 (0.61 – 0.80)	0.67 (0.59 – 0.77)
Site area		1.51 (1.30 – 1.76)	1.59 (1.36 – 1.86)		1.58 (1.36 – 1.85)
Area of non-emergent vegetation	1.96 (1.68 – 2.29)				
Area of mud	1.00 (0.85 – 1.18)				

	Model 1	***Model 2***	***Model 3***	***Model 4***	***Model 5***
Predictors	***Incidence rate ratio (CI)***	***Incidence rate ratio (CI)***	***Incidence rate ratio (CI)***	***Incidence rate ratio (CI)***	***Incidence rate ratio (CI)***
Percent cover of non-emergent vegetation		1.67 (1.44 – 1.95)	1.57 (1.34 – 1.84)		1.56 (1.33 – 1.84)
Percent cover of mud		1.05 (0.92 – 1.20)	1.05 (0.92 – 1.19)		1.24 (1.04 – 1.47)
Mass of non-emergent vegetation			1.24 (1.06 – 1.46)	1.90 (1.56 – 2.33)	1.33 (1.11 – 1.60)
Snail density					0.89 (0.60 – 1.32)
Snail prevalence					1.28 (1.06 – 1.54)
**Random effects**					
σ^2	1.20	1.19	1.19	1.19	1.19
τ_{00}	2.28 $_{\text{ID:village}}$	2.30 $_{\text{ID:village}}$	2.31 $_{\text{ID:village}}$	2.15 $_{\text{ID:village}}$	2.29 $_{\text{ID:village}}$
	0.00 $_{\text{village}}$	0.00 $_{\text{village}}$	0.00 $_{\text{village}}$	0.17 $_{\text{village}}$	0.00 $_{\text{village}}$

***SI Appendix,* Table S13.** A non-exhaustive list of references documenting associations between schistosome-competent snails and non-emergent vegetation.

**Country**	**Snail species**	**Schistosome species**	**Non-emergent vegetation taxon**	**Reference**
Morocco	*Bulinus truncatus*	*Schistosoma haematobium*	*Potamogeton* sp.	(41)
Egypt and Sudan	*Biomphalaria pfeifferi*	*Schistosoma mansoni*	Many species	(42)
Egypt and Sudan	*Bulinus spp.*	*Schistosoma haematobium*	Many species	(42)
Ghana	*Bulinus* sp.	*Schistosoma haematobium*	*Ceratophyllum* sp.	(43)
Ghana	*Bulinus truncatus rohlfsi*	*Schistosoma haematobium*	*Ceratophyllum* sp.	(44)
Ghana	*Bulinus truncatus rohlfsi*	*Schistosoma haematobium*	*Ceratophyllum* sp.	(45)
Ghana	*Bulinus truncatus rohlfsi* and *Bulinus globosus*	*Schistosoma haematobium*	*Ceratophyllum* sp.	(46)
Ghana	*Bulinus truncatus rohlfsi*	*Schistosoma haematobium*	*Ceratophyllum* sp.	(47)
Ghana	*Bulinus truncatus rohlfsi*	*Schistosoma haematobium*	*Ceratophyllum* sp.	(48)
Ghana	*Bulinus* sp.	*Schistosoma haematobium*	*Nymphaea* sp.	(43)
Ghana	*Bulinus truncatus rohlfsi*	*Schistosoma haematobium*	*Nymphaea* sp.	(48)
Ghana	*Bulinus truncatus rohlfsi*	*Schistosoma haematobium*	*Pistia* sp.	(44)
Ghana	*Bulinus truncatus rohlfsi*	*Schistosoma haematobium*	*Pistia* sp.	(45)
Ghana	*Bulinus truncatus rohlfsi*	*Schistosoma haematobium*	*Salvinia* sp.	(44)
Ghana	*Bulinus truncatus rohlfsi*	*Schistosoma haematobium*	*Salvinia* sp.	(45)
Nigeria	*Bulinus truncatus rohlfsi*	*Schistosoma haematobium*	*Ceratophyllum* sp.	(49)
Nigeria	*Bulinus truncatus rohlfsi*	*Schistosoma haematobium*	*Salvinia* sp.	(49)
Nigeria	*Biomphalaria pfeifferi*	*Schistosoma mansoni*	*Ceratophyllum* sp.	(49)
Nigeria	*Biomphalaria pfeifferi*	*Schistosoma mansoni*	*Salvinia* sp.	(49)
Kenya	*Bulinus nasutus*	*Schistosoma haematobium*	*Nymphaea* sp.	(50)
Zimbabwe	*Bulinus globosus*	*Schistosoma haematobium*	*Nymphaea* sp.	(51)
Zimbabwe	*Biomphalaria pfeifferi*	*Schistosoma mansoni*	*Nymphaea* sp.	(51)
Brazil	*Biomphalaria glabrata*	*Schistosoma mansoni*	Species not identified	(52)
Brazil	*Biomphalaria glabrata* and *B. straminea*	*Schistosoma mansoni*	Species not identified	(53)
Brazil	*Biomphalaria* spp.	*Schistosoma mansoni*	“submergent and emergent vegetation”	(54)

Supporting Information References

1. Thorson J (2017) Three problems with the conventional delta-model for biomass sampling data, and a computationally efficient alternative. *Canadian Journal of Fisheries and Aquatic Science* 75:1369-1382.
2. Ortu G, *et al.* (2017) Countrywide reassessment of *Schistosoma mansoni* infection in Burundi using a urine-circulating cathodic antigen rapid test: Informing the national control program. *American Journal of Tropical Medicine and Hygiene* 96:664-673.
3. Assare RK, *et al.* (2016) Sustaining control of schistosomiasis mansoni in western Côte d'Ivoire: Results from a SCORE Study, one year after initial praziquantel administration. *PLoS Neglected Tropical Diseases* 10:e0004329.
4. Karanja DMS, *et al.* (2017) Cluster randomized trial comparing school-based mass drug administration schedules in areas of western Kenya with moderate initial prevalence of *Schistosoma mansoni* infections. *PLoS Neglected Tropical Diseases* 11:e0006033.
5. Phillips AE, *et al.* (2017) Assessing the benefits of five years of different approaches to treatment of urogenital schistosomiasis: A SCORE project in Northern Mozambique. *PLoS Neglected Tropical Diseases* 11:e0006061.
6. Olsen A, Kinung'hi S, and Magnussen P. (2018) Comparison of the impact of different mass drug administration strategies on infection with *Schistosoma mansoni* in Mwanza Region, Tanzania—A cluster-randomized controlled trial. *American Journal of Tropical Medicine and Hygiene* 99:1573-1579.
7. Knopp S, *et al.* (2019) A 5-Year intervention study on elimination of urogenital schistosomiasis in Zanzibar: Parasitological results of annual crosssectional surveys. *PLoS Neglected Tropical Diseases* 13:e0007268.
8. Kittur N, *et al.* (2019) Persistent hotspots in Schistosomiasis Consortium for Operational Research and Evaluation studies for gaining and sustaining control of schistosomiasis after four years of mass drug administration of praziquantel. *American Journal of Tropical Medicine and Hygiene* 101:617-627.
9. Planet Labs, https://www.planet.com. Accessed 11 Sep 2019.
10. Digital Globe Foundation, http://foundation.digitalglobe.com. Accessed 11 Sep 2019.
11. Southgate V*, et al.* (2000) Observations on the compatibility between *Bulinus* spp. and *Schistosoma haematobium* in the Senegal River basin. *Annals of Tropical Medicine and Parasitology* 94(2):157-164.
12. Sene M, Southgate V, & Vercruysse J (2004) *Bulinus truncatus*, intermediate host of *Schistosoma haematobium* in the Senegal River Basin (SRB). *Bulletin de la Societe de Pathologie Exotique* 97(1):29-32.
13. Huyse T*, et al.* (2009) Bidirectional introgressive hybridization between a cattle and human schistosome species. *PloS Pathogens* 5(9):e1000571.
14. Kane RA, Stothard J, Emery A, & Rollinson D (2008) Molecular characterization of freshwater snails in the genus *Bulinus*: A role for barcodes? *Parasites and Vectors* 1:15.
15. Abdel Malek E (1958) Factors conditioning the habitat of bilharziasis intermediate hosts of the family Planorbidae. *Bulletin of the World Health Organization* 18:785-818.
16. Thomas J & Tait A (1984) Control of the snail hosts of schistosomiasis by environmental manipulation: A field and laboratory appraisal in the Ibadan Area, Nigeria. *Philosophical Transactions of the Royal Society B* 305:201-253.
17. Klumpp RK & Chu KY (1977) Ecological studies of *Bulinus rohlfsi*, the intermediate host of *Schistosoma haematobium* in the Volta Lake. *Bulletin of the World Health Organization* 55(6):715-730.
18. McCullough F (1956) Transmission of *Schistosoma haematobium* by *Bulinus* sp. in the Ke District of the Gold Coast. *Transactions of the Royal Society of Tropical Medicine and Hygiene* 50(5):449-457.

19. Paperna I (1970) Study of an outbreak of schistosomiasis in the newly formed Volta Lake in Ghana. *Zeitschrift fur Tropenmedizin und Parasitologie* 21(4):411-425.
20. Paperna I (1969) Aquatic weeds, snails, and transmission of bilharzia in the new man-made Volta Lake in Ghana. *Bulletin de l'Institut Fondamental d'Afrique Noire, Serie A. Sciences Naturelles* 31(A):487-499.
21. Klumpp R & Chu K (1980) Importance of the aquatic weed *Ceratophyllum* to transmission of *Schistosoma haematobium* in the Volta Lake, Ghana. *Bulletin of the World Health Organization* 58(5):791-798.
22. Odei M (1973) Observations of some weeds of malacological importance in the Volta Lake in Ghana. *Bulletin de l'Institut Francais d'Afrique Noire* 31 Ser A(2):57-66.
23. Woolhouse ME & Chandiwana SK (1989) Spatial and temporal heterogeneity in the population dynamics of *Bulinus globosus* and *Biomphalaria pfeifferi* and in the epidemiology of their infection with schistosomes. *Parasitology* 98:21-34.
24. Kariuki HC*, et al.* (2004) Distribution patterns and cercarial shedding of *Bulinus nasutus* and other snails in the Msambweni area, Coast Province, Kenya. *American Journal of Tropical Medicine and Hygiene* 70(4):449-456.
25. Zhou Y-B*, et al.* (2016) Multi-host model and threshold of intermediate host *Oncomelania* snail density for eliminating schistosomiasis transmission in China. *Nature Scientific Reports* 6:31089.
26. Gower C*, et al.* (2007) Development and application of an ethically and epidemiologically advantageous assay for the multi-locus microsatellite analysis of *Schistosoma mansoni*. *Parasitology* 134:523-536.
27. Webster B*, et al.* (2015) Development of novel multiplex microsatellite polymerase chain reactions to enable high-throughput population genetic studies of *Schistosoma haematobium*. *Parasites and Vectors* 8(1):432.
28. Webster BL, Diaw OT, Seye M, Webster JP, & Rollinson D (2013) Introgressive hybridization of *Schistosoma haematobium* group species in Senegal: Species barrier break down between ruminant and human schistosomes. *PLoS Neglected Tropical Diseases* 7(4):e2110.
29. Emery A, Allan F, Rabone M, & Rollinson D (2012) Schistosomiasis Collection at NHM (SCAN). *Parasites and Vectors* 5:185.
30. Folmer O, Black M, Hoeh W, Lutz R, & Vrijenhoek R (1994) DNA primers for amplification of mitochondrial cytochrome c oxidase subunit I from diverse metazoan invertebrates. *Molecular Marine Biology and Biotechnology* 3(5):294-299.
31. Kulldorff M & Information Management Services I (2009) SaTScanTM v8.0: Software for the spatial and space-time scan statistics.
32. Kulldorff M (2015) *SaTScan User Guide for version 9.4*.
33. Kulldorff M, Heffernan R, Hartman J, Assunção R, & Mostashari F (2005) A space-time permutation scan statistic for the early detection of disease outbreaks. *PLoS Medicine* 2:216-224.
34. Ribeiro SHR & Costa MA (2012) Optimal selection of the spatial scan parameters for cluster detection: A simulation study. *Spatial and Spatio-Temporal Epidemiology* 3:107-120.
35. Ma Y, Yin F, Zhang T, Zhou XA, & Li X (2016) Selection of the maximum spatial cluster size of the spatial scan statistic by using the maximum clustering set-proportion statistic. *PLoS One* 11(1):e0147918.
36. Kulldorff M*, et al.* (2004) Benchmark data and power calculations for evaluating disease outbreak detection methods. *Morbidity and Mortality Weekly Report* 53:144-151.
37. Venables W & Ripley B (2002) *Modern Applied Statistics with S: Fourth Edition* (Springer, New York, NY).

38. Thorson JT, Shelton AO, Ward EJ, and Skaug HJ (2015) Geostatistical delta-generalized linear mixed models improve precision for estimated abundance indices for West Coast groundfishes. *ICES Marine Science* 72:1297-1310.
39. Horton NJ and Kleinman KP (2007) Much ado about nothing: A comparison of missing data methods and software to fit incomplete data regression models. *American Statistician* 61:79-90.
40. Kristensen K, Nielsen A, Berg CW, Skaug H, and Bell B (2015) TMB: Automatic Differentiation and Laplace Approximation. *Journal of Statistical Software* 70:1-21.
41. Boelee E & Laamrani H (2004) Environmental control of schistosomiasis through community participation in a Moroccan oasis. *Tropical Medicine and International Health* 9(9):997-1004.
42. Abdel Malek E (1958) Factors conditioning the habitat of bilharziasis intermediate hosts of the family Planorbidae. *Bulletin of the World Health Organization* 18:785-818.
43. McCullough F (1956) Transmission of *Schistosoma haematobium* by *Bulinus* sp. in the Ke District of the Gold Coast. *Transactions of the Royal Society of Tropical Medicine and Hygiene* 50(5):449-457.
44. Paperna I (1969) Aquatic weeds, snails, and transmission of bilharzia in the new man-made Volta Lake in Ghana. *Bulletin de l'Institut Fondamental d'Afrique Noire, Serie A. Sciences Naturelles* 31(A):487-499.
45. Paperna I (1970) Study of an outbreak of schistosomiasis in the newly formed Volta Lake in Ghana. *Zeitschrift fur Tropenmedizin und Parasitologie* 21(4):411-425.
46. Odei M (1973) Observations of some weeds of malacological importance in the Volta Lake in Ghana. *Bulletin de l'Institut Francais d'Afrique Noire* 31 Ser A(2):57-66.
47. Klumpp RK & Chu KY (1977) Ecological studies of *Bulinus rohlfsi*, the intermediate host of *Schistosoma haematobium* in the Volta Lake. *Bulletin of the World Health Organization* 55(6):715-730.
48. Klumpp R & Chu K (1980) Importance of the aquatic weed *Ceratophyllum* to transmission of *Schistosoma haematobium* in the Volta Lake, Ghana. *Bulletin of the World Health Organization* 58(5):791-798.
49. Thomas J & Tait A (1984) Control of the snail hosts of schistosomiasis by environmental manipulation: A field and laboratory appraisal in the Ibadan Area, Nigeria. *Philosophical Transactions of the Royal Society B* 305:201-253.
50. Kariuki HC*, et al.* (2004) Distribution patterns and cercarial shedding of *Bulinus nasutus* and other snails in the Msambweni area, Coast Province, Kenya. *American Journal of Tropical Medicine and Hygiene* 70(4):449-456.
51. Woolhouse M & Chandiwana S (1989) Spatial and temporal heterogeneity in the population dynamics of *Bulinus globosus* and *Biomphalaria pfeifferi* and in the epidemiology of their infection with schistosomes. *Parasitology* 98:21-34.
52. Kloos H, Passos L, LoVerde P, Oliveira R, & Gazzinelli A (2004) Distribution and *Schistosoma mansoni* infection of *Biomphalaria glabrata* in different habitats in a rural area in the Jequitinhonha Valley, Minas Gerais, Brazil: Environmental and epidemiological aspects. *Memorias do Instituto Oswaldo Cruz* 99(7):673-681.
53. Kloos H*, et al.* (2001) The distribution of *Biomphalaria* spp. in different habitats in relation to physical, biological, water contact and cognitive factors in a rural area in Minas Gerais, Brazil. *Memorias do Instituto Oswaldo Cruz* 96:57-66.
54. Bavia ME, Hale LF, Malone JB, Braud DH, & Shane SM (1999) Geographic information systems and the environmental risk of schistosomiasis in Bahia, Brazil. *The American Journal of Tropical Medicine and Hygiene* 60:566-572.
